# ALPL regulates pro-angiogenic capacity of mesenchymal stem cells through ATP-P2X7 axis controlled exosomes secretion

**DOI:** 10.1186/s12951-024-02396-6

**Published:** 2024-04-12

**Authors:** Jiayi Dong, Wanmin Zhao, Jiangdong Zhao, Ji Chen, Ping Liu, Xueni Zheng, Dehua Li, Yang Xue, Hongzhi Zhou

**Affiliations:** 1 State Key Laboratory of Oral & Maxillofacial Reconstruction and Regeneration, National Clinical Research Center for Oral Diseases, Shaanxi Clinical Research Center for Oral Diseases, Department of Oral Surgery, School of Stomatology, The Fourth Military Medical University, Xi’an, China; 2 State Key Laboratory of Oral & Maxillofacial Reconstruction and Regeneration, National Clinical Research Center for Oral Diseases, Shaanxi International Joint Research Center for Oral Diseases, Center for Tissue Engineering, School of Stomatology, The Fourth Military Medical University, Xi’an, China; 3 The Key Laboratory of Aerospace Medicine, Ministry of Education, Air Force Medical University, Xi’an, China; 4 State Key Laboratory of Oral & Maxillofacial Reconstruction and Regeneration, National Clinical Research Center for Oral Diseases, Shaanxi Engineering Research Center for Dental Materials and Advanced Manufacture, Department of Oral Implants, School of Stomatology, The Fourth Military Medical University, Xi’an, China

**Keywords:** Hypophosphatasia, Angiogenesis, BMMSCs, Exosomes, ALPL, P2X7

## Abstract

**Background:**

Early-onset bone dysplasia is a common manifestation of hypophosphatasia (HPP), an autosomal inherited disease caused by ALPL mutation. ALPL ablation induces prototypical premature bone ageing characteristics, resulting in impaired osteogenic differentiation capacity of human bone marrow mesenchymal stem cells (hBMMSCs). As angiogenesis is tightly coupled with osteogenesis, it also plays a necessary role in sustaining bone homeostasis. We have previously observed a decrease in expression of angiogenesis marker gene CD31 in the metaphysis of long bone in *Alpl*^+/−^ mice. However, the role of ALPL in regulation of angiogenesis in bone has remained largely unknown.

**Methods:**

Exosomes derived from Normal and HPP hBMMSCs were isolated and identified by ultracentrifugation, transmission electron microscopy, and nanoparticle size measurement. The effects of ALPL on the angiogenic capacity of hBMMSCs from HPP patients were assessed by immunofluorescence, tube formation, wound healing and migration assay. exo-ELISA and Western Blot were used to evaluate the exosomes secretion of hBMMSCs from HPP, and the protein expression of VEGF, PDGFBB, Angiostatin and Endostatin in exosomes respectively.

**Results:**

We verified that ALPL ablation resulted in impaired pro-angiogenic capacity of hBMMSCs, accounting for reduced migration and tube formation of human umbilical vein endothelial cells, as the quantities and proteins composition of exosomes varied with ALPL expression. Mechanistically, loss of function of ALPL enhanced ATP release. Additional ATP, in turn, led to markedly elevated level of ATP receptor P2X7, which consequently promoted exosomes secretion, resulting in a decreased capacity to promote angiogenesis. Conversely, inhibition of P2X7 increased the angiogenic induction capacity by preventing excessive release of anti-angiogenic exosomes in ALPL deficient-hBMMSCs.

**Conclusion:**

The ALPL–ATP axis regulates the pro-angiogenic ability of hBMMSCs by controlling exosomes secretion through the P2X7 receptor. Thus, P2X7 may be proved as an effective therapeutic target for accelerating neovascularization in ALPL–deficient bone defects.

**Supplementary Information:**

The online version contains supplementary material available at 10.1186/s12951-024-02396-6.

## Introduction

Tissue nonspecific alkaline phosphatase (TNSALP) is a cellular enzyme that is encoded by the ALPL gene in liver, bone, and kidney [1, 2]. Loss of function of the ALPL gene results in hypophosphatasia (HPP), a hereditary disease with high incidence. HPP is characterized by loss of bone mass and decreased bone density and can lead to osteoporosis, pathological fracture, and skeletal deformities over time. However, as osteogenesis is coupled with angiogenesis in bone repair and regeneration [3], increasing the capacity for angiogenesis can promote restoration of physiologic homeostasis in the damaged tissues. In our previous study, ALPL deficiency not only impaired the lineage differentiation of human bone marrow mesenchymal stem cells (hBMMSCs) and weakened osteogenesis [1, 4, 5], but also inhibited blood vessel formation in the metaphysis of long bone in *Alpl*^+/−^ mice [6]. However, the exact mechanism by which ALPL regulates the angiogenesis ability of hBMMSCs has remained poorly defined, including whether the regulation is direct or indirect. Thus, we aim herein to elucidate the pathogenic mechanism, with the aim to provide new insights into bone defect repair and regeneration for precision treatment of the disease.

Mesenchymal stem cells (MSCs) possess highly proliferative and multilineage differentiation potential and coordinate with the vascular system to maintain homeostasis of the bone environment [7, 8]. Physiologically, molecular crosstalk between MSCs and endothelial cells (ECs) provides a paracrine environment to activate ECs and promotes all processes of angiogenesis [9]. Exosomes, which are important mediators of the communication between MSCs and ECs, are membrane vesicles secreted by various types of cells and appealing candidates as vectors of MSCs efficacy [10]. They are created by budding at both plasma and endosome membranes, have the same topology as the cell, and are enriched in certain proteins, lipids, and nucleic acids [11, 12]. Exosomes have multifaceted roles in modulation of various biological responses, including the angiogenic regulation of MSCs’ crosstalk with ECs [13, 14]. It has been suggested that MSCs-derived exosomes have been suggested to play an active part in blood vessel development and progression in the repair of multiple tissue types [15, 16]. Angiogenesis is critical in maintaining bone homeostasis and promoting bone regeneration; however, it remains unclear whether MSCs can affect newly blood vessels formation through exosomal signalling and lead to pathological changes in HPP patients.

The quantity and quality of exosomes vary between physiological and pathological conditions, which can be regulated by genetic manipulation of the parental MSCs and potentially inverte the fate of the target cells completely [17, 18]. Aberrant exosomes secretion is associated with various human diseases, including Parkinson’s disease [19], multiple sclerosis [20], and Alzheimer’s disease [21]. Adenosine triphosphate (ATP) is a highly versatile extracellular molecule that has been implicated in a variety of cellular activities, from energy provision to cell-to-cell communication [22]. P2X7 as an ATP-gated cation channel, the activation of which has been proved to not only promote secretion of extracellular vesicles, but also alter the content of exosomes [23]. It has been confirmed that P2X7 promotes metastatic spreading by enhancing production of miRNA-containing exosomes and microvesicles from melanoma cells [24]. Our previous study proved that ALPL deficiency in MSCs could result in redundancy ATP accumulation, exhibited the fate switch in MSCs differentiation and senescence [4]. However, whether this excessive ATP could account for aberrant exosomes secretion in MSCs and impaired cell–cell communication with ECs by regulating the P2X7 has not been determined.

In the present study, hBMMSCs with a loss-of-function mutation in the ALPL gene were obtained from HPP patients. Investigation of the effects of ALPL on cell–cell communication between hBMMSCs and ECs suggested that ALPL promoted the pro-angiogenic capability of hBMMSCs via boosting of hBMMSCs–ECs communication. Then, we investigated the manner by which ALPL in hBMMSCs regulated the angiogenic potential of ECs and observed that ALPL mediated the pro-angiogenic capacity of hBMMSCs by an exosomes pathway. Specifically, we focused that ALPL altered the quantity and protein composition of exosomes, which may contribute to explaining the angiogenic potential induction of hBMMSCs. Mechanistically, we revealed that the ALPL–ATP axis regulated exosomes secretion of hBMMSCs by the P2X7 receptor. Hence, the results of the current study provide new insights, including that the ALPL–ATP axis plays an essential role in regulation of new blood vessel formation during bone regeneration through regulating release of exosomes, suggesting a need for further exploration of the function of the ALPL gene and the potential for new treatment methods via the revaluation ALPL.

## Materials and methods

### Human subjects

Two HPP patients (male) aged 8 years were treated by the Affiliated Hospital of the Fourth Military Medical University for osteodynia. The healthy human bone marrow (BM) samples were collected from three teenagers aged 12–14 years (male) who underwent the bone defect in an open lower limb fracture with bone grafting. The clinical study was approved by the Ethics Committee of the Fourth Military Medical University (IRB-REV-2013-002), and informed consent was obtained from all parental/legal guardian prior to tissue collection. It also conducted in accordance with the guidelines outlined in the Declaration of Helsinki.

### Isolation and culture of hBMMSCs

Cells were purified from BM using the Percoll density gradient centrifugation method and cultured in alpha minimum essential medium (α-MEM, Gibco, 41090036, USA) with 10% fetal basal medium (FBS, Gibco, 10099141, USA), 2 mmol·L^−1^ L-glutamine, 100 U·mL^−1 ^penicillin and 100 mg·mL^−1^ streptomycin (Invitrogen, 15140148, USA) at 37 ℃ in 5% CO_2_ condition. After 48 h, non-adherent cells were washed with phosphate buffered saline (PBS, Gibco, 10010023, USA) and the attached cells were passaged with trypsin containing 1mM EDTA after being cultured for 14 days.

### Wound healing assay

For investigating the paracrine effect of hBMMSCs on ECs migration, 3 × 10^4^ hBMMSCs were seeded into upper chamber of 6-well Corning Transwell plates (an 0.4 μm pore size; Corning, USA). Then, 5 × 10^4^ ECs were then seeded in the lower chamber. Following growth to 100% confluence, cells were subjected to single vertical scratches using a 200 µL pipette tip. The upper chamber of the transwell, which were already seeded with hBMMSCs, were washed three times with PBS and then co-cultured with ECs, and images were recorded at 0 and 12 h after scratching. To investigate the effect of hBMMSCs exosomes on ECs migration, ECs were seeded in 6-well plates, with hBMMSCs derived exosomes (25 µg/mL) were added. Images were then recorded at 0 and 12 h after scratching using an optical microscope (Lecia, Germany). The rate of wound closure was calculated as follows: (mean wound width-mean remaining width) / mean wound width × 100%.

### Migration assay

Transwell migration assays were used to determine the effects of hBMMSCs derived exosomes on the ability to recruit ECs. Briefly, 3 × 10^4^ ECs were seeded in the upper chamber of 24-well transwell plates containing a membrane of pore size 8 μm in each well. hBMMSCs exosomes (25 µg/mL) were added to the upper chamber containing serum-free medium. 500 µL of conditioned medium was placed in the lower chamber. Migrated cells were cultured for 6 h and then fixed for 15 min, followed by crystal violet staining for 10 min. Non- migrating cells in the upper chambers were gently removed using cotton swabs, and cells on the outside of the filters were counted and photographed by an optical microscope (Lecia, Germany).

### Tube formation assay

The tube formation assay were conducted using the Matrigel basement membrane matrix (Corning, 356234, USA) according to the manufacture’s instructions. To study the paracrine effect of hBMMSCs on tube formation, they were co-cultured with ECs using 24-well transwell plates containing a 0.4 μm pore size membrane in each well. Firstly, approximately 3 × 10^4^ hBMMSCs were seeded in an upper chamber with 500 µL medium containing exosomes-deprived FBS. After 48 h, Matrigel was added to the lower chamber about 300 µL per well using ice-cold tips and incubated at 37 ℃ for solidification. Then, 1.2 × 10^5^ ECs were seeded into solidified Matrigel and co-cultured with hBMMSCs. After 3 h incubation, the cells were photographed using an inverted optical microscope (Lecial, Germany) and the number of tube formation and branch length was calculated by ImageJ software. To examine the effect of hBMMSCs exosomes to induce new vessels formation, Matrigel was dissolved and 96-well plates were coated. ECs were seeded on Matrigel-coated wells and cultured in medium supplemented with 1% FBS and hBMMSCs exosomes (25 µg/mL). Cells were then incubated for 3 h and photographed using an inverted optical microscope (Lecial, Germany).

### Exosomes extraction and characterization

After hBMMSCs had grown to approximately 80% fusion, the culture medium was replaced with exosomes-deprived medium. After an additional 48 h culture, the medium was collected and firstly centrifuged at 2000 g for 10 min. The supernatant was then centrifuged at 10,000 g for 30 min. After that, the supernatant was ultracentrifuged at 100,000 g for 70 min. The pellet was finally washed with PBS and ultracentrifuged at 100,000 g for a further 70 min again. The exosomes at the bottom of the tube were then resuspended in PBS. To identify exosomes, the morphology of exosomes was detected using a Thermo Fisher transmission electron microscopy (TEM; Thermo, USA). Nanoparticle tracking analysis (NTA) was used to determine the size of exosomes. For analysis of hBMMSCs exosomes secretion. Western Blot was used to compare the content of exosomes proteins from equal volumes of culture supernatant from different hBMMSCs. Exosomes ELISA complete kit (SBI, USA) was used to quantify vesicles number, following the manufacturer’s instruction [22].

### Exosomes uptake assay

hBMMSCs derived exosomes was labeled with PKH26 fluorescence dye (Sigma-Aldrich, USA) according to the manufacturer’s protocol. ECs were then incubated with PKH26-labelled exosomes for 12 h. After fixation with 4% paraformaldehyde for 15 min, cells were blocked with 5% BSA for 20–30 min, followed by staining with a solution of fluorescent phalloidin in PBS for 20 min at room temperature. Nuclei were counterstained using DAPI for 10 min. Lastly, the uptake phenomenon was observed using a laser scanning confocal microscope (Nikon, Japan).

### Quantification of angiogenic protein by ELISA

The secreted VEGF, PDGFBB, Angiostatin and Endostatin in the samples were quantified using human VEGF ELISA Kit (Proteintech, KE00216, China), human PDGFBB ELISA Kit (Proteintech, KE00161, China), human Angiostatin ELISA kit (Neobioscience Technology, E-EL-H6165, China) and human Endostatin ELISA Kit (Proteintech, KE00259, China) according to manufacture’s instruction. The level of VEGF, PDGFBB, Angiostatin and Endostatin was determined at 450 nm.

### Measurement of ATP concentration

The hBMMSCs were collected by ATP lysis byffer and centrifuged at 12,000 g at 4 ℃ for 5 min; then, the supernatants of the hBMMSCs were examined by an Enhanced ATP Assay Kit (Beyotime, China).

### Immunofluorescent staining

hBMMSCs were seeded onto chamber slides (Nunc, USA) (4 × 10^4^/well), and cells were fixed with 4% PFA. Primary antibodies (1:100) were added to the chamber slides and incubated at 4℃ overnight. After being washed in PBS three times, the slides were treated with secondary antibodies (1:200) for 75 min at room temperature. Positive cells were examined using a laser scanning confocal microscope (Nikon, Japan).

### Western blot

hBMMSCs were harvested in RIPA lysis buffer (Beyotime Co, Shanghai, China, P0013B). After the whole-cell protein extracts were quantified by a bicinchoninic acid (BCA), extracts were separated on SDS polyacrylamide gel electrophoresis (PAGE) and transferred onto polyvinylidene fluoride (PVDF) membranes (Millipore, Billerica, MA, USA). After blocking with 5% bovine serum albumin (BSA, MP Biomedicals, USA) for 1 h at room temperature, the membranes were incubated overnight with primary antibodies (1:1000). After being washed in Tris-buffered saline-Tween (TBST, solarbio, China, T1081), the membranes were incubated in secondary antibody (1:10,000) for 1 h. Lastly, signals were visualized using electrochemiluminescence (ECL) analysis (Tanon-Bio, 4600) and quantified by ImageJ software.

### Antibodies

The antibody to ALPL (11187-1-AP) was purchased from Proteintech (Wuhan, China). Antibody to CD81 (GTX31381) was purchased from GeneTex (Texas, USA). Antibodies to CD9 (ab92726), TSG101 (ab125011), PDGFBB (ab16829) and P2X7 (ab48871) were purchased from Abcam (Cambridge, USA). Antibodies to VEGF (SC-57496) and Endostatin (SC-32720) were purchased from Santa Cruz Biotechnology (Santa Cruz, CA, USA). Antibody to Angiostatin (AF-226) was purchased from R&D (R&D sysstems, USA). Anti β-Tubulin antibody was purchased from Cwbiobiotech (Beijing, China).

### Lentiviral vector construction and transduction

ALPL was amplified form human genomic DNA by PCR in order to construct a lentiviral vector expressing the human *ALPL*. The PCR product was digested with BamH I and Xho I restriction enzymes, inserted into the pLenti 6.3 vector (Invitrogen, USA), named pLenti-ALPL. For downregulated ALPL, shRNA sequences targeting to human ALPL were inserted into pLko.1 vector (Invitrogen, USA). To produce lentivirus, 293T cells were co-transfected with the transfer vector and two packaging vectors (i.e., psPAX2 and pMD2.G). Subsequently, the virus was purified using ultracentrifugation. hBMMSCs were plated in 75cm^2^ cultured bottles and transducted with lentiviral constructs and 5 µg/mL polybrene (Sigma, USA, 107689). Primers used to construct the lentiviral vectors of ALPL are listed in supplementary Table 1.

### Transfection assay

siRNA duplex oligonucleotides against human P2X7 were obtained from Ribo (GuangZhou, China). Non-targeting control siRNAs were used as negative controls. The siRNAs were then transfected into the cells at a final concentration of 50 nM using a RiboFect CP Transfection Kit according to the manufacturer’s instructions. The medium was replaced after 8 h.

### Statistics and reproducibility

All data were presented as mean ± standard deviation (SD). Prior to further analysis, the data were evaluated for normal distributions and similar variances between groups. Unpaired, two-tailed Student’s t-test was used for two groups comparisons and one-way analysis of variance (ANOVA) with Bonferroni for multiple comparisons. P values less than 0.05 were considered to be significant. Despite the highly variable nature of the studies performed, the size of all the experimental groups were chosen to ensure adequate statistical power.

## Results

### ALPL deficiency in hBMMSCs impairs angiogenesis through paracrine pathway

**Fig. 1:**
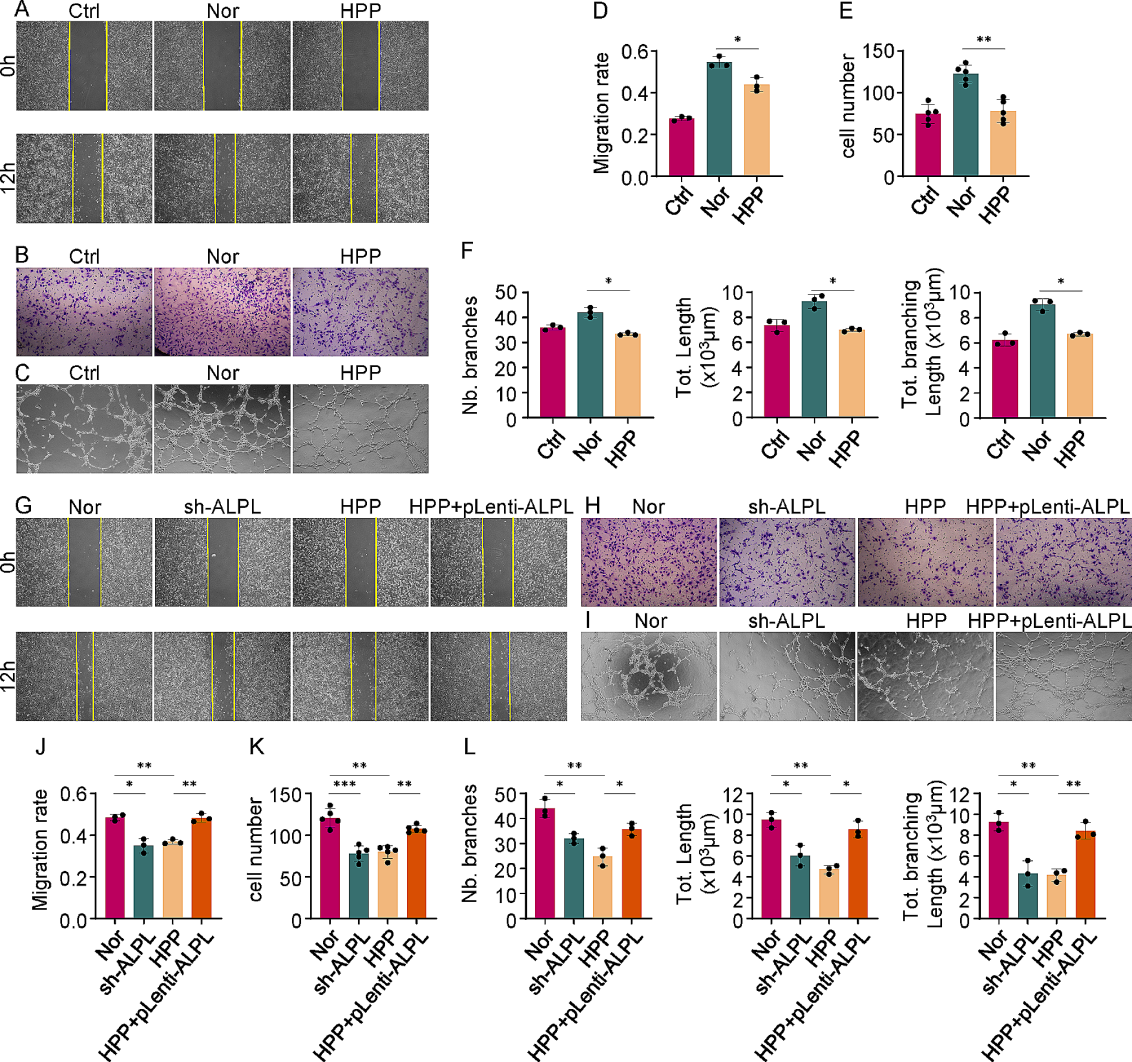
ALPL deficiency in hBMMSCs inhibits ECs angiogenesis by paracrine pathway. **(A)** The migration rate of ECs co-cultured with Nor and HPP hBMMSCs, as assessed by scratch wound healing assay at 0 and 12 h. **(B)** The migration ability of ECs, as assessed by migration assay after 6 h of co-culture. **(C)** The tube formation capacity of ECs, as assessed by tube formation assay after 3 h of co-culture. (**D-F)** The quantitative assay for scratch wound healing assay **(D)** migration assay **(E)** and tube formation assay **(F)** respectively. ECs cultured with medium was served as the control group (Ctrl). To evaluate whether ALPL in hBMMSCs could induce ECs angiogenesis, we upregulated or downregualted the expression of ALPL in HPP hBMMSCs with pLenti-ALPL (HPP + pLenti-ALPL) or Normal hBMMSCs with ALPL shRNA (sh-ALPL) lentiviral transduction. **(G)** The migration rate of ECs co-cultured with Nor, sh-ALPL, HPP and HPP + pLenti-ALPL hBMMSCs, as assessed by scratch wound healing assay at 0 and 12 h. **(H)** The migration ability of ECs, as assessed by migration assay after 6 h of co-culture. **(I)** The tube formation capacity of ECs, as assessed by tube formation assay after 3 h of co-culture. (**J-L)** The quantitative assay for scratch wound healing assay **(J)** migration assay **(K)** and tube formation assay **(L)** respectively. All results were generated in three independent experiments. Data were shown as mean ± standard deviation (SD); *P < 0.05; **P < 0.01; ***P < 0.001

ALPL gene deletion leads to decreased osteogenic differentiation ability of BMMSCs [4, 5]. Given that bone regeneration relies on the coupling of osteogenesis and angiogenesis [3, 25, 26], we speculated whether deletion of the ALPL gene would affect the formation of new vessels in bone. First, hBMMSCs with characteristics typical of MSCs were successfully isolated (Additional file 1: Fig. S1A-C). The transwell co-culture models were applied to detect the paracrine effects of MSCs on ECs. Using a scratch wound healing assay, we found that co-culture of HPP-patient-derived hBMMSCs (HPP hBMMSCs) and ECs significantly slowed the ECs migration rate to scratched regions at 12 h, compared with co-culture of normal-individual-derived hBMMSCs (Nor hBMMSCs) and ECs. However, the scratched areas in both the Nor hBMMSCs and HPP hBMMSCs groups were significantly smaller than that of the ECs alone (control group) at each time point (Fig. 1A and D). In the cell migration analysis, a significant decrease in the number of migrated ECs was observed after culture with HPP hBMMSCs compared with those cultured with Nor hBMMSCs (Fig. 1B and E). The tube formation assay showed that the branch numbers, total tube length, and branching length were increased in the co-culture groups compared to the control group. Consistent with the results of the cell migration assays, the capacity for promoting tube formation of the HPP hBMMSCs group was lower than that of the Nor hBMMSCs group (Fig. 1C and F).

To further examine the angiogenic role of ALPL in hBMMSCs, we used lentiviral transduction to downregulate ALPL in Nor hBMMSCs (sh-ALPL) and upregulate ALPL in HPP hBMMSCs (HPP + pLenti-ALPL) (Additional file 1: Fig. S1D and E). sh-ALPL hBMMSCs led to a significant reduction of ECs migration rate in the co-culture assay, whereas, the ability of HPP + pLenti-ALPL hBMMSCs to induce migration of ECs was restored (Fig. 1G and J). Similarly, the sh-ALPL hBMMSCs reduced the number of migrated ECs, mimicking the HPP hBMMSCs phenotype. Conversely, the HPP + pLenti-ALPL hBMMSCs rescued the cell migration (Fig. 1H and K). Finally, the tube formation assay also showed that the sh-ALPL hBMMSCs inhibited the tube formation of ECs, whereas the HPP + pLenti-ALPL hBMMSCs rescued the impairment in angiogenesis capacity, as confirmed by observation of tube formation numbers, branch lengths, and total length (Fig. 1I and L). These data demonstrate that the angiogenic-promoting effects of MSCs on ECs are regulated by paracrine, which are reduced by ALPL mutation.

### Ability of ALPL to regulate pro-angiogenic capacity of hBMMSCs is blocked by GW4869

**Fig. 2:**
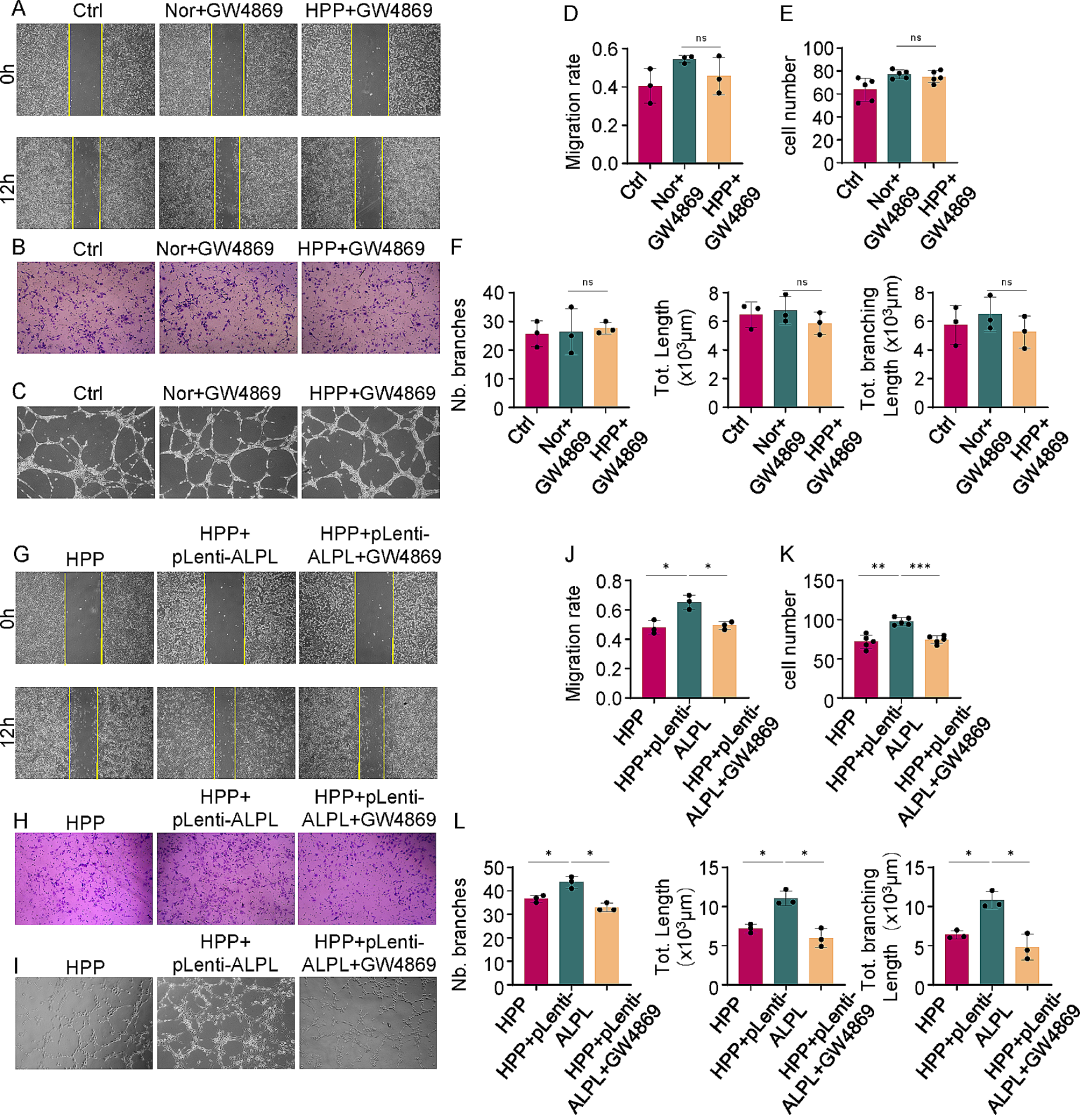
Inhibition of exosomes secretion by GW4869 blocks pro-angiogenic ability in the ALPL deficient hBMMSCs. **(A)** The migration rate of ECs after treatment with GW4869 pre-treated Nor hBMMSCs (Nor + GW4869) and HPP hBMMSCs (HPP + GW4869), as assessed by scratch wound healing assay. **(B)** The migration ability of ECs, as assessed by migration assay after 6 h of co-culture. **(C)** The tube formation capacity of ECs, as assessed by tube formation assay after 3 h of co-culture. (**D-F)** The quantitative assay for scratch wound healing assay **(D)** migration assay **(E)** and tube formation assay **(F)** respectively. ECs cultured with medium was served as the control group (Ctrl). **(G)** The migration rate of ECs after treatment with HPP, HPP + pLenti-ALPL and GW4869 pre-treated HPP + pLenti-ALPL hBMMSCs (HPP + pLenti-ALPL + GW4869), as assessed by the wound healing assay at 0 and 12 h. **(H)** The migration ability of ECs, as assessed by migration assay after 6 h of co-culture. **(I)** The tube formation capacity of ECs, as assessed by tube formation assay after 3 h of co-culture. (**J-L)** The quantitative assay for scratch wound healing assay **(J)** migration assay **(K)** and tube formation assay **(L)** respectively. All results were generated in three independent experiments. Data were shown as mean ± standard deviation (SD); *P < 0.05; **P < 0.01; ***P < 0.001

The formation and biological activity of exosomes can be highly regulated under different physiological and pathological conditions [27–29]. To determine whether ALPL’s angiogenic regulation of ECs is depended on the hBMMSCs’ exosomes pathway, we used GW4869 to block secretion of exosomes from Nor and HPP hBMMSCs prior to co-culture with ECs. The migration rate and tube formation numbers of ECs co-cultured with Nor hBMMSCs showed no statistically significant difference compared with those cultured with HPP hBMMSCs (Fig. 2A–F). To further confirm these findings, we also blocked exosomes secretion of HPP + pLenti-ALPL hBMMSCs. Consistently, the angiogenic capacity restored by ALPL gene overexpression was inhibited (Fig. 2G-L). Collectively, these results indicate that ALPL in hBMMSCs regulates the angiogenic ability of ECs by secreting exosomes.

### ALPL mutation inhibits pro-angiogenic capacity through hBMMSCs exosomes

**Fig. 3:**
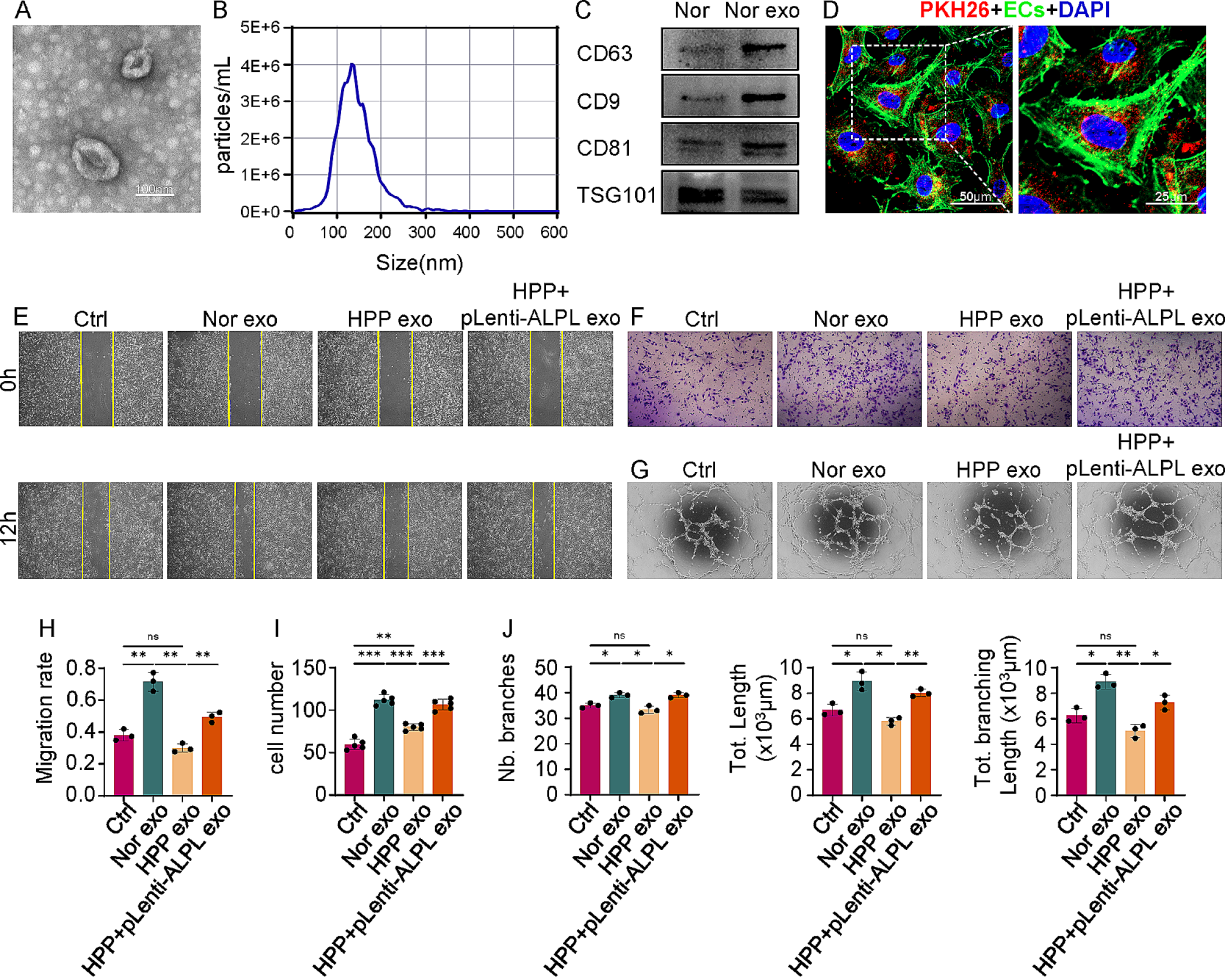
**ALPL deficiency inhibits the pro-angiogenesis ability of hBMMSCs exosomes. (A)** Transmission electron microscopy exhibited the typical morphology of hBMMSCs exosomes. Scale bar, 100 nm. **(B)** Nanoparticle size distribution of hBMMSCs exosomes. **(C)** Western Blot analysis of exosomal proteins including CD63, CD9, CD81 and TSG101. **(D)** The uptake of hBMMSCs exosomes by ECs. ECs were incubated with PKH26-lablled exosomes (PKH26-exo; PKH26 is shown in red). ECs were co-stained with phalloidin (green) and nuclei were stained with DAPI (blue). Scale bar 50 μm. Magnification image of the area marked by white box was shown in right panel. Scale bar 25 μm. **(E)** The migration rate of ECs cultured in different types of exosomes at equal concentrations (25 µg/mL), as assessed by scratch wound healing assay at 0 and 12 h. **(F)** The migration ability of ECs, as assessed by migration assay after 6 h of culture. **(G)** The tube formation capacity of ECs, as assessed by tube formation assay after 3 h of culture. (**H-J)** The quantitative assay for scratch wound healing assay **(H)** migration assay **(I)** and tube formation assay **(J)** respectively. ECs cultured with medium was served as the control group (Ctrl). All results were generated in three independent experiments. Data were shown as mean ± standard deviation (SD); *P < 0.05; **P < 0.01; ***P < 0.001

Next, we identified features of exosomes derived from Normal (Nor exo) and HPP hBMMSCs (HPP exo) respectively, which were isolated and characterized by transmission electron microscopy analysis (TEM), nanoparticle tracking analysis (NTA) and Western Blot. TEM revealed typically round, bi-layer membrane-bound vesicles (Fig. 3A and Additional file 1: Fig. S1F) ranging in size from 100 to 200 nm in diameter and NTA exhibited a similar size distribution in borth of the Nor and HPP exo (Fig. 3B and Additional file 1: Fig. S1G). Western Blot revealed the expression of exosomes surface markers including CD63, CD9, CD81 and TSG101 in Nor and HPP exo respectively (Fig. 3C and Additional file 1: Fig. S1H). In addition, we found that exosomes labelled with fluorescent red dye PKH26 could be internalized by ECs (Fig. 3D).

We then investigated the effects of ALPL on regulation of the angiogenic potential of hBMMSCs exo. ECs were treated with Nor, HPP and HPP + pLenti-ALPL exo, which derived from HPP + pLenti-ALPL hBMMSCs. Nor exo enhanced the migration rate of ECs compared with HPP exo or control groups. Furthermore, HPP + pLenti-ALPL exo also promoted the ECs migration rate compared with HPP exo (Fig. 3E, F, H and I). According to the results of the tube formation assay, Nor exo efficiently promoted the tube like structures formation of ECs, including markedly increased number of branches, as well as overall length and branch length compared with those of ECs alone or HPP exo groups. Furthermore, compared with HPP exo, HPP + pLenti-ALPL exo also efficiently promoted the formation of tube-like structures of ECs (Fig. 3G and J). Taken together, these data demonstrate that ALPL affects the angiogenic function of ECs by regulating hBMMSCs exosomes.

### ALPL controls exosomes secretion in hBMMSCs

**Fig. 4:**
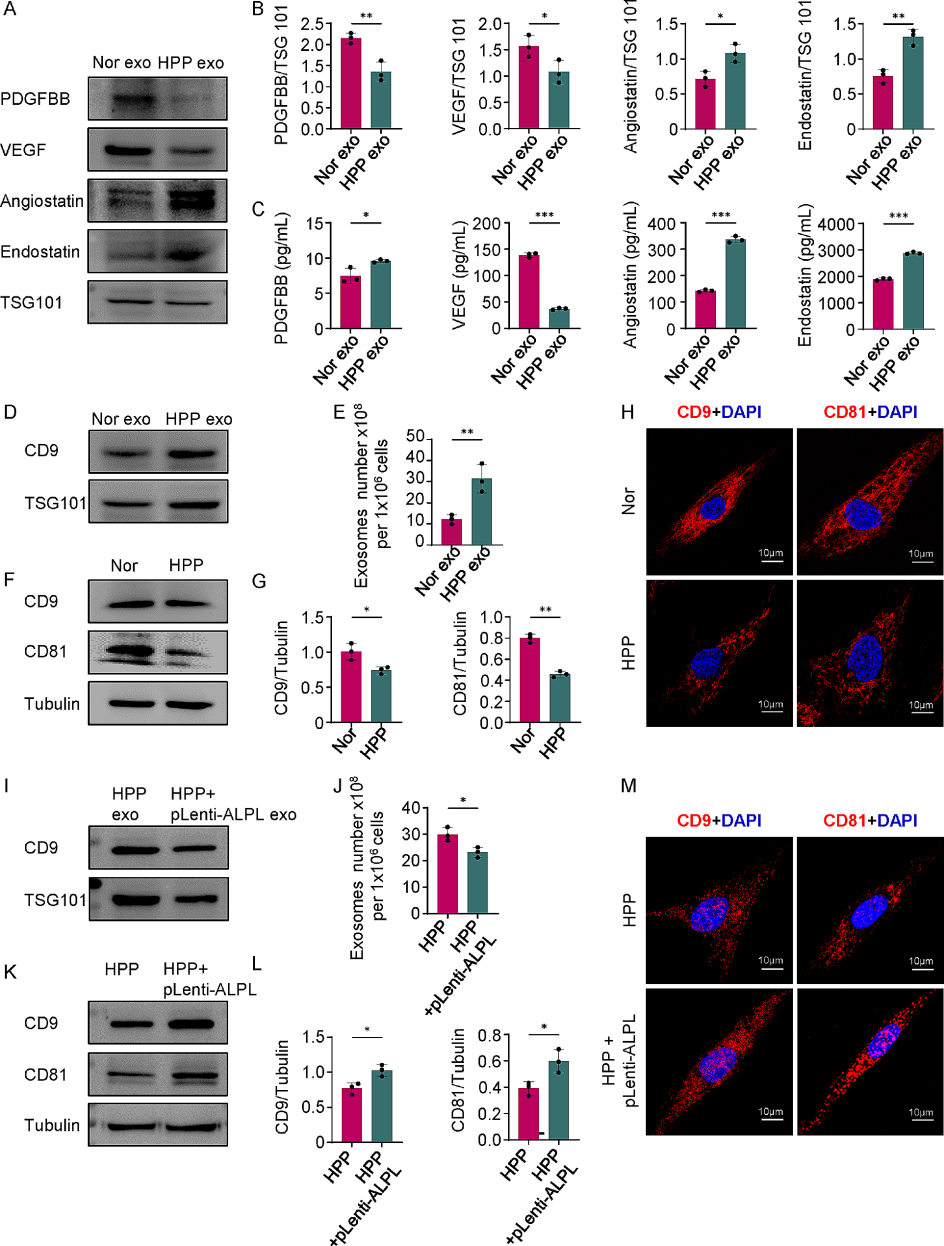
ALPL deficiency induces excessive anti-angiogenic exosomes secretion in hBMMSCs. **(A-B)** Western Blot analysis showed PDGFBB, VEGF, Angiostatin and Endostatin expression in Nor and HPP exosomes. **(C)** ELISA analysis of PDGFBB, VEGF, Angiostatin and Endostatin levels in the Nor and HPP group. **(D)** Western Blot showed exosomes from Nor and HPP hBMMSCs expressed CD9 and TSG101. Exosomes proteins from equal volumes of culture supernatant of Nor and HPP hBMMSCs were loaded for Western Blot. (**E)** Exosomes volumes derived from Nor and HPP hBMMSCs were detected by exosomes ELISA complete kit. **(F-G)** The intracellular expression of CD9 and CD81 were assessed by Western Blot analysis. **(H)** Immunofluorescent staining showed CD9 and CD81-positive labeled exosomal proteins localized in Nor and HPP hBMMSCs. Scale bar, 10 μm. **(I)** Western Blot showed exosomes from HPP and HPP + pLenti-ALPL hBMMSCs expressed CD9 and TSG101. Exosomes proteins from equal volumes of culture supernatant of HPP and HPP + pLenti-ALPL hBMMSCs were loaded for Western Blot. **(J)** Exosomes volumes derived from HPP and HPP + pLenti-ALPL hBMMSCs were detected by exosomes ELISA complete kit. **(K-L)** The intracellular expression of CD9 and CD81 were assessed by Western Blot analysis. **(M)** Immunofluorescent staining showed CD9 and CD81-positive labeled exosomal proteins localized in HPP and HPP + pLenti-ALPL hBMMSCs. Scale bar, 10 μm. All results were generated in three independent experiments. Data were shown as mean ± standard deviation (SD); *P < 0.05; **P < 0.01; ***P < 0.001

As exosomes contain a variety of proteins, we analyzed the expression levels of pro-angiogenic and anti-angiogenic proteins in various exosomes. Western Blot analysis confirmed that the level of pro-angiogenic proteins VEGF (vascular endothelial growth factor) and PDGFBB (platelet-derived growth factor BB) were downregulated by more than half in the HPP exo group compared with the Nor exo group. However, anti-angiogenic proteins Angiostatin and Endostatin were markedly upregulated in HPP exo (Fig. 4A and B). ELISA assay also indicated that the level of pro-angiogenic proteins VEGF and PDGFBB were also reduced and anti-angiogenic proteins Angiostatin and Endostatin were elevated in HPP exo (Fig. 4C). To further elucidate the effects of anti-angiogenic exosomes released from hBMMSCs under conditions of ALPL deficiency, we upregulated (pLenti-ALPL) and downregulated (sh-ALPL) ALPL gene expression by lentiviral transfection of Nor hBMMSCs (Additional file 1: Fig. S2A and B). Pro-angiogenic proteins were surprisingly upregulated in exosomes derived from pLenti-ALPL hBMMSCs (pLenti-ALPL exo) compared with Nor exo. By contrast, anti-angiogenic proteins were strikingly downregulated in pLenti-ALPL exo but upregulated in the exosomes of sh-ALPL hBMMSCs (sh-ALPL exo) (Additional file 1: Fig. S2C and D). ELISA assay was also performed the quantity of pro- and anti-angiogenic proteins in different exosomes, which was consistent with Western Blot (Additional file 1: Fig. S2E). Collectively, these results indicate that ALPL regulates the angiogenic protein content of hBMMSCs exosomes, which in turn serve as crucial regulators of ECs’ function and angiogenesis.

In addition to protein composition [28], the quantity of exosomes [30] has been previously verified to have an important effect on the cellular behaviour. Here, we found that exosomes derived from HPP hBMMSCs expressed high levels of CD9 and TSG101 compared with Nor hBMMSCs (Fig. 4D). Using an exosomes ELISA quantitation kit, we also confirmed that exosomes secretion was increased in HPP hBMMSCs (Fig. 4E). Then, to further evaluate whether ALPL could regulate exosomes secretion, we upregulated and downregulated ALPL in hBMMSCs by lentivirus transfection. Western Blot demonstrated that exosomes markers CD9 and TSG101 were highly expressed in sh-ALPL hBMMSCs, consistent with their levels in HPP hBMMSCs (Additional file 1: Fig. S3A). Application of the exosomes ELISA quantitation kit showed that more exosomes were secreted in the sh-ALPL group than that in the normal group (Additional file 1: Fig. S3B). Given that HPP + pLenti-ALPL hBMMSCs rescued the capacity of ECs angiogenesis, we hypothesized that ALPL overexpression in HPP hBMMSCs may reduce aberrant exosomes secretion. As predicted, compared with the HPP hBMMSCs, the excessive exosomes release were prevented in HPP + pLenti-ALPL hBMMSCs, as evidenced by the Western Blot and exosomes ELISA quantitation kit (Fig. 4I and J).

To further confirm this phenomenon, we examined intracellular exosomal proteins (CD9 and CD81) and found that the accumulation of exosomes in HPP hBMMSCs was significantly reduced compared with that in Nor hBMMSCs (Fig. 4F and G). Immunofluorescence staining showed that fewer exosomal proteins accumulated in HPP hBMMSCs (Fig. 4H). Next, we tested intracellular exosomes accumulation in the normal, pLenti-ALPL, and sh-ALPL hBMMSCs groups; similarly, there were fewer exosomes in the downregulation group, whereas more intracellular exosomes accumulated in pLenti-ALPL hBMMSCs compared with Nor hBMMSCs (Additional file 1: Fig. S3C-E). More convincingly, HPP + pLenti-ALPL hBMMSC exhibited a stronger capacity to increase the intracellular exosomal proteins accumulation than HPP hBMMSCs as evidenced by higher expression levels of CD9 and CD81 by Western Blot and immunofluorescence staining (Fig. 4K-M). These results suggest that ALPL is a crucial regulator of exosomes secretion in hBMMSCs.

#### ALPL deficiency in hBMMSCs increases exosomes secretion via ATP axis

**Fig. 5:**
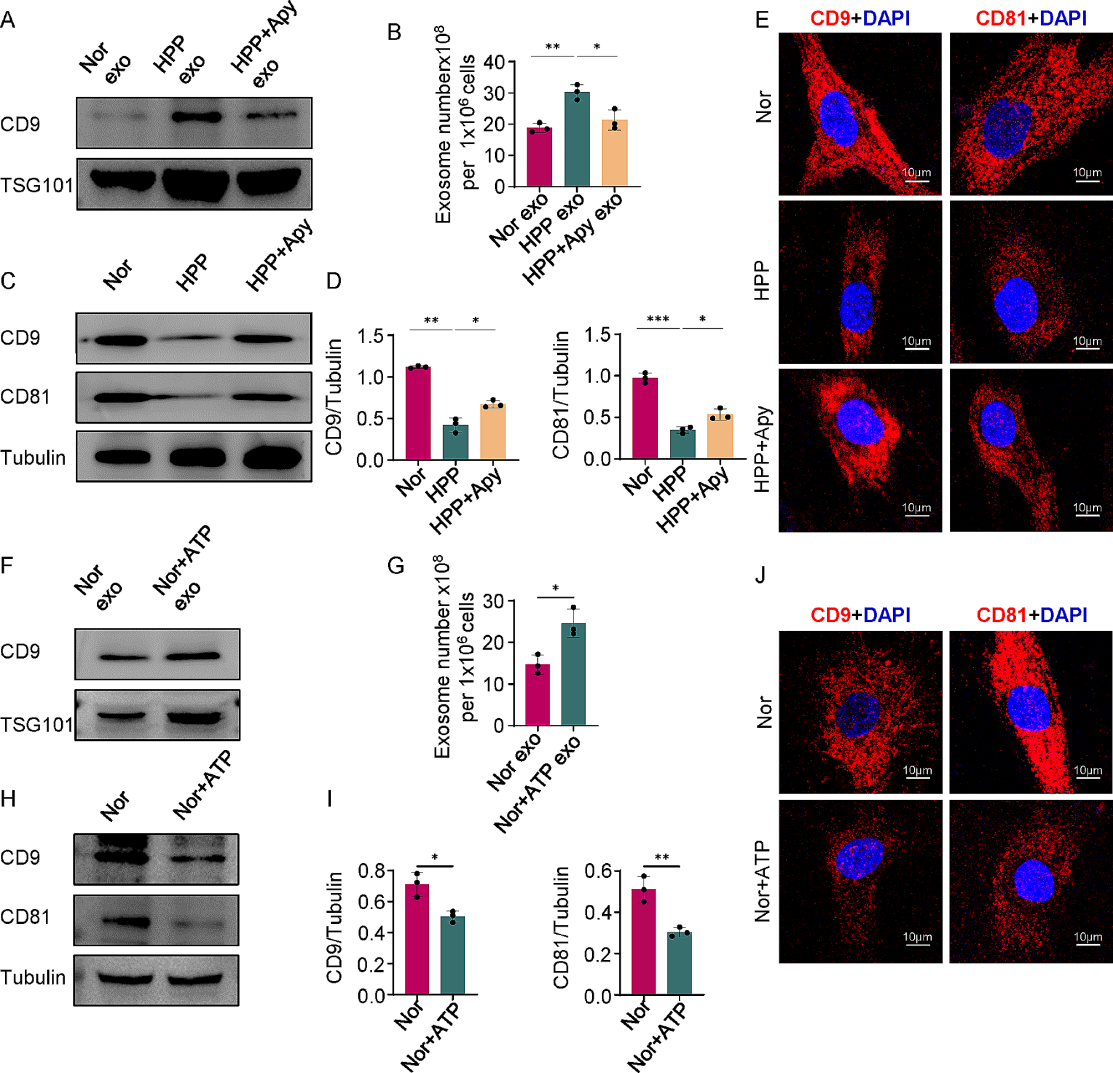
ALPL mutation increases hBMMSCs exosomes secretion through ATP axis. **(A)** HPP hBMMSCs was treated with 2 U/mL ATP-apyrase (HPP + Apy) and the exosomes markers were analyzed. Exosomes proteins from equal volumes of culture supernatant of Nor, HPP and HPP + Apy hBMMSCs were loaded for Western Blot. **(B)** Exosomes volumes derived from each groups were detected by exosomes ELISA complete kit. **(C-D)** The intracellular expression of CD9 and CD81 were analyzed by Western Blot. **(E)** Immunofluorescent staining showed CD9 and CD81-positive labeled exosomal proteins localized in Nor, HPP and HPP + Apy hBMMSCs. Scale bar, 10 μm. **(F)** Nor hBMMSCs was treated with 10µmol/L ATP and the exosomes markers were analyzed. Exosomal proteins from equal volumes of culture supernatant of Nor and Nor + ATP hBMMSCs were loaded for Western Blot. **(G)** Exosomes volumes derived from Nor and Nor + ATP groups were detected by exosomes ELSA complete kit. **(H-I)** The intracellular expression of CD9 and CD81 were analyzed by Western Blot. **(J)** CD9 and CD81-positive labeled exosomal proteins localized in Nor and Nor + ATP hBMMSCs, as assessed by immunofluorescent staining. Scale bar, 10 μm. All results were generated in three independent experiments. Data were shown as mean ± standard deviation (SD); *P < 0.05; **P < 0.01; ***P < 0.001

Next, we explored the pathway by which ALPL regulated the secretion of hBMMSCs exosomes. Previous studies have shown that deletion of the ALPL gene in hBMMSCs leads to increased intracellular and extracellular ATP concentrations, which induces senescence of hBMMSCs by inhibiting osteogenesis and promoting adipogenesis [4, 5]. Hence, we surmised that the increased secretion of exosomes would also be regulated by the ALPL–ATP axis. Consistently, we also confirmed the extracellular ATP level in the HPP hBMMSCs was higher than that in the Normal hBMMSCs medium and the excessively high level of extracellular ATP concentration was also detected in the sh-ALPL hBMMSCs, suggesting that ALPL in MSCs regulates ATP release (Additional file 1: Fig. S4A and B). Subsequently, we used ATP apyrase to hydrolyze an excess of ATP in HPP hBMMSCs (HPP + Apy hBMMSCs). Western Blot and ELISA quantification kit indicated that decreased ATP level led to a reduction in exosomes secretion compared with HPP hBMMSCs (Fig. 5A and B). Moreover, ATP hydrolysis resulted in a greater accumulation of intracellular exosomes in HPP + Apy hBMMSCs compared with HPP hBMMSCs, as evidenced by Western Blot showing that intracellular exosomal protein levels in the HPP + Apy group were higher than those in the HPP group (Fig. 5C and D). Immunofluorescence staining indicated that large numbers of CD9-positive and CD81-positive intracellular exosomes accumulated in HPP + Apy hBMMSCs (Fig. 5E).

Conversely, addition of exogenous ATP to Nor hBMMSCs (Nor + ATP hBMMSCs) increased exosomes secretion compared with that of the normal group, even without any change in ALPL gene expression (Fig. 5F and G). Similarly, fewer intracellular exosomes accumulated in Nor + ATP hBMMSCs compared with Nor hBMMSCs, as determined by Western Blot and immunofluorescence staining (Fig. 5H-J). Taken together, these data demonstrate that ALPL regulates the exosomes secretion of hBMMSCs via the ATP axis, clarifying that there is a tight link between the ALPL–ATP axis and exosomes secretion.

### ALPL–ATP axis regulates exosomes secretion potential of hBMMSCs via P2X7 receptor

**Fig. 6:**
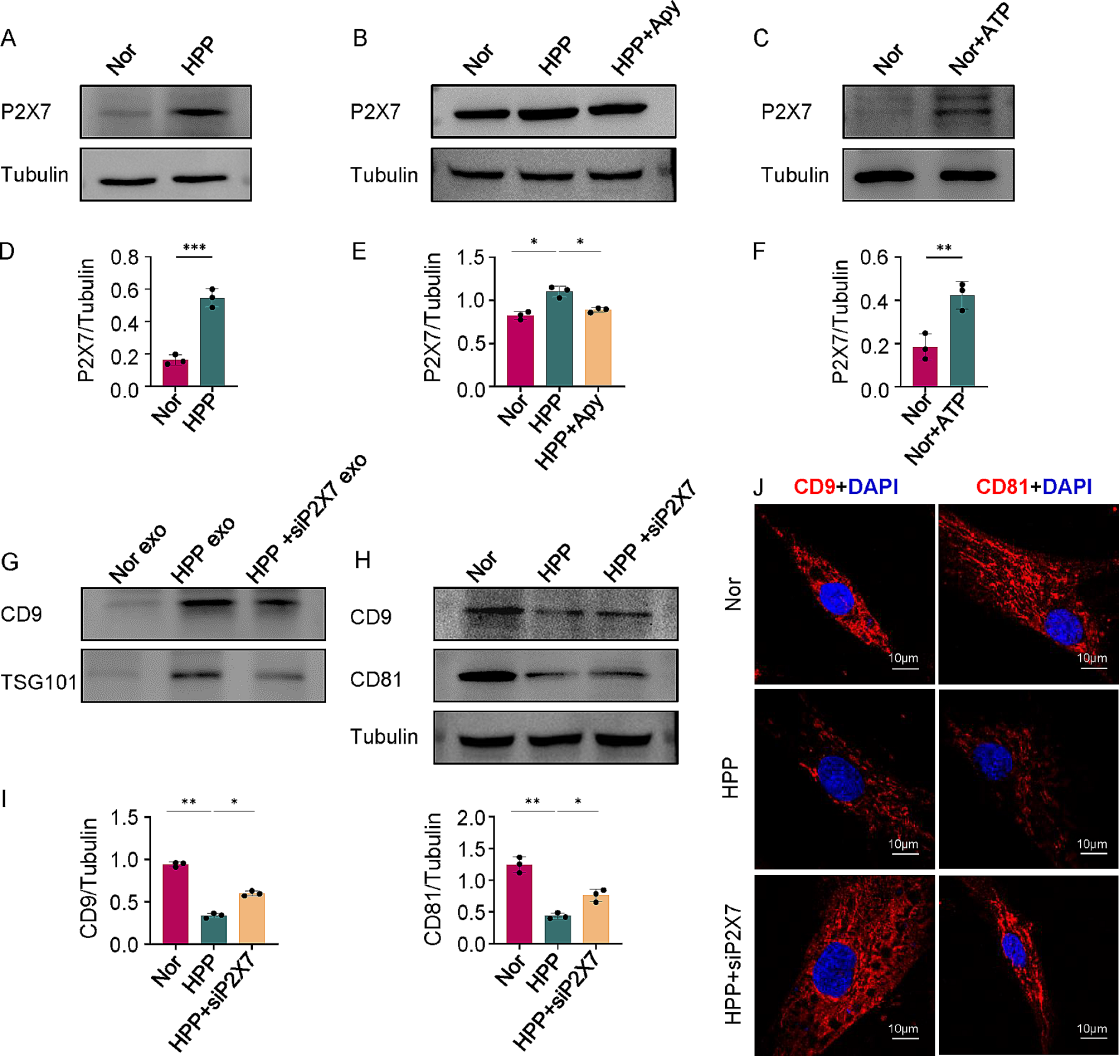
P2X7 serves as a key regulator of ALPL-ATP axis mediated exosomes secretion. **(A, D)** The expression of P2X7 in Nor and HPP hBMMSCs, as assessed by Western Blot analysis. **(B, E)** The expression of P2X7 was analyzed by Western Blot in Nor, HPP and HPP + Apy hBMMSCs. **(C, F)** The expression of P2X7 was analyzed by Western Blot in Nor and Nor + ATP hBMMSCs. **(G)** P2X7 siRNA-treated HPP hBMMSCs (HPP + siP2X7) was collected and exosomes marker were analyzed. Exosomal proteins from equal volumes of culture supernatant of Nor, HPP and HPP + siP2X7 hBMMSCs were loaded for Western Blot. **(H-I)** The intracellular expression of CD9 and CD81 were examined by Western Blot. **(J)** CD9 and CD81-positive labeled exosomal proteins localized in Nor, HPP and HPP + siP2X7 hBMMSCs. Scale bar, 10 μm. All results were generated in three independent experiments. Data were shown as mean ± standard deviation (SD); *P < 0.05; **P < 0.01; ***P < 0.001

ATP regulates cellular function through activation of ionotropic P2X receptors and metabotropic P2Y receptors [30]. P2X7 is an important ATP receptor that can control exosomes release and exosomes secretion is positively correlated with P2X7 expression [23]. To identify the mechanism involved in the secretion of hBMMSCs exosomes, we examined the relationship between the ALPL-ATP axis and P2X7. The results showed that P2X7 expression was increased in HPP hBMMSCs comparing to Nor hBMMSCs (Fig. 6A and D). Similar results were obtained in sh-ALPL hBMMSCs, however, pLenti-ALPL hBMMSCs inhibited P2X7 expression (Additional file 1: Fig. S4C and D). To further examine the effects of ALPL on P2X7, we added apyrase to HPP hBMMSCs, which resulted in a decrease in ATP levels and inhibited P2X7 expression, suggesting that the abnormally high ATP levels in HPP may stimulate expression of P2X7 (Fig. 6B and E). By contrast, the addition of exogenous ATP to Nor hBMMSCs resulted in an increase in P2X7 expression compared with Nor hBMMSCs (Fig. 6C and F). These data indicate that ALPL exerts a certain regulatory effect on the expression of P2X7 in hBMMSCs through the ATP axis, and that this pathway may have a significant role in the regulation of exosomes secretion.

To investigate the role of P2X7 in exosomes secretion of hBMMSCs, we used short interfering RNA (siRNA) to knock down the expression of P2X7 in HPP hBMMSCs (HPP + siP2X7 hBMMSCs) and sh-ALPL hBMMSCs (sh-ALPL + siP2X7 hBMMSCs) (Additional file 1: Fig. S4E-H). As well as P2X7 inhibition, reduced secretion of abnormal extracellular exosomes was also observed in HPP + siP2X7 hBMMSCs, as evidenced by a decrease in expression levels of CD9 and TSG101 (Fig. 6G). Western Blot and immunofluorescence staining indicated increased accumulation of intracellular exosomal proteins in HPP + siP2X7 hBMMSCs compared with HPP hBMMSCs (Fig. 6H–J). Furthermore, similar to the findings in HPP + siP2X7 hBMMSCs, siRNA knockdown of P2X7 in sh-ALPL hBMMSCs reduced the secretion of excessive exosomes and increased the accumulation of intracellular exosomes (Additional file 1: Fig. S4I-L). Hence, these data show that the ALPL–ATP axis is required to maintain exosomes release from hBMMSCs through regulation of P2X7 levels.

#### P2X7 inhibition rescues angiogenesis impairment through reducing excessive anti-angiogenic exosomes release in the ALPL-deficient hBMMSCs

**Fig. 7:**
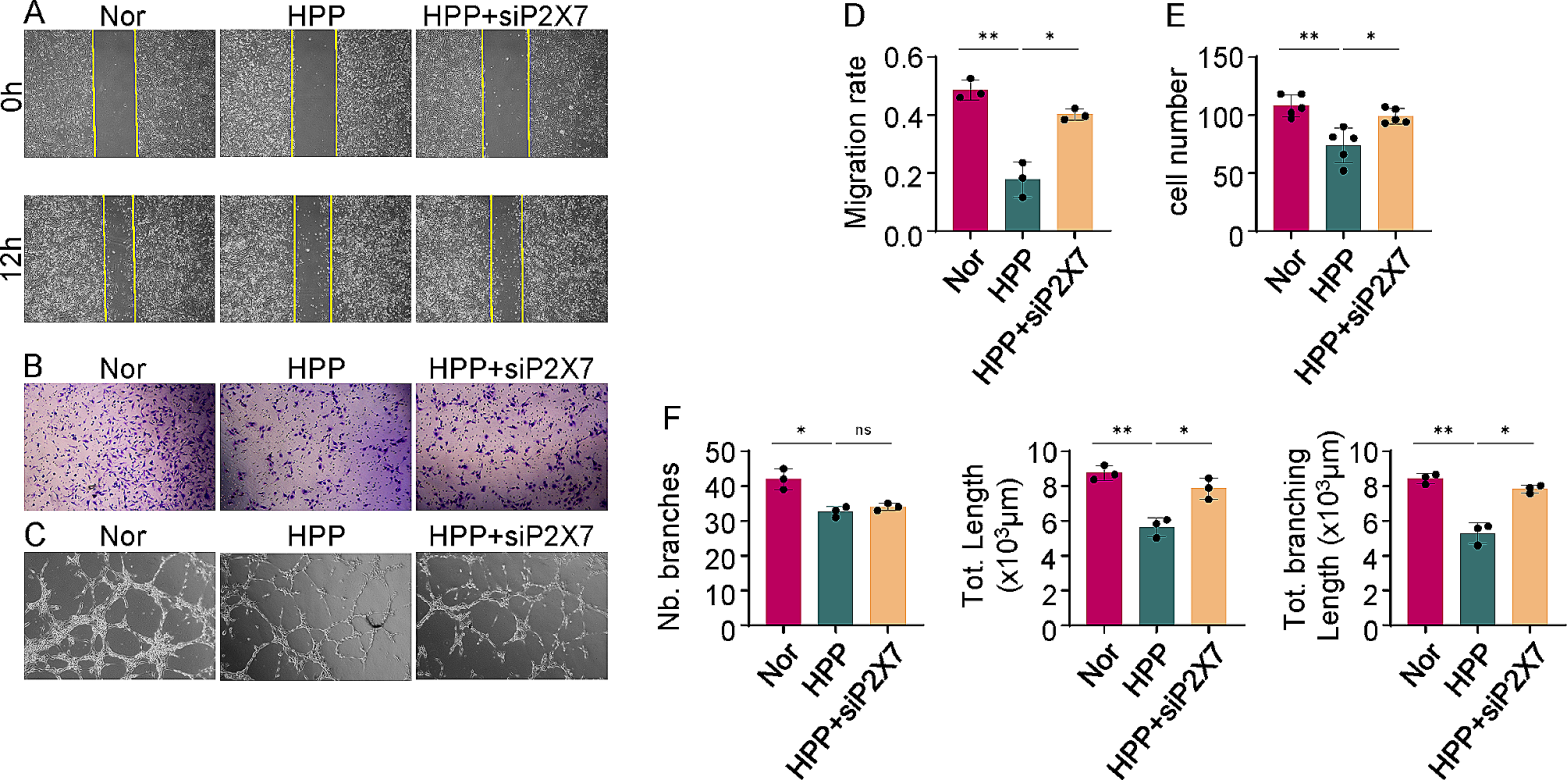
P2X7 inhibition restores the pro-angiogenic capacity of HPP hBMMSCs. **(A)** The migration rate of ECs co-cultured with Nor, HPP and HPP + siP2X7 hBMMSCs, as assessed by scratch wound healing assay at 0 and 12 h. **(B)** The migration ability of ECs, as assessed by migration assay after 6 h of co-culture. **(C)** The tube formation capacity of ECs, as assessed by tube formation assay after 3 h of co-culture. (**D-F)** The quantitative assay for scratch wound healing assay **(D)** migration assay **(E)** and tube formation assay **(F)** respectively. All results were generated in three independent experiments. Data were shown as mean ± standard deviation (SD); *P < 0.05; **P < 0.01; ***P < 0.001

To further determine whether P2X7 could regulate angiogenesis through hBMMSCs exosomes, we performed knockdown of P2X7 in HPP hBMMSCs using siRNA. Scratch wound healing and cell migration assays confirmed that ECs co-cultured with HPP + siP2X7 hBMMSCs migrated more rapidly than those co-cultured with HPP hBMMSCs (Fig. 7A, B, D and E). The tube formation assay revealed that the formation of tube-like structures, branches numbers, and overall length were all rescued in ECs co-cultured with HPP + siP2X7 hBMMSCs (Fig. 7C and F). These observations suggest that P2X7 can regulate angiogenesis through the control of exosomes release. Consistent with this view, according to the results of the scratch wound healing and cell migration assays, the migration rate of ECs was also significantly increased when they were co-cultured with sh-ALPL + siP2X7 hBMMSCs (Additional file 1: Fig. S5A, B, D and E). Similarly, tube-like structures, branching length, and total length of ECs co-cultured with sh-ALPL + siP2X7 hBMMSCs were found to be increased in comparison with those in the sh-ALPL hBMMSCs group (Additional file 1: Fig. S5C and F).

**Fig. 8:**
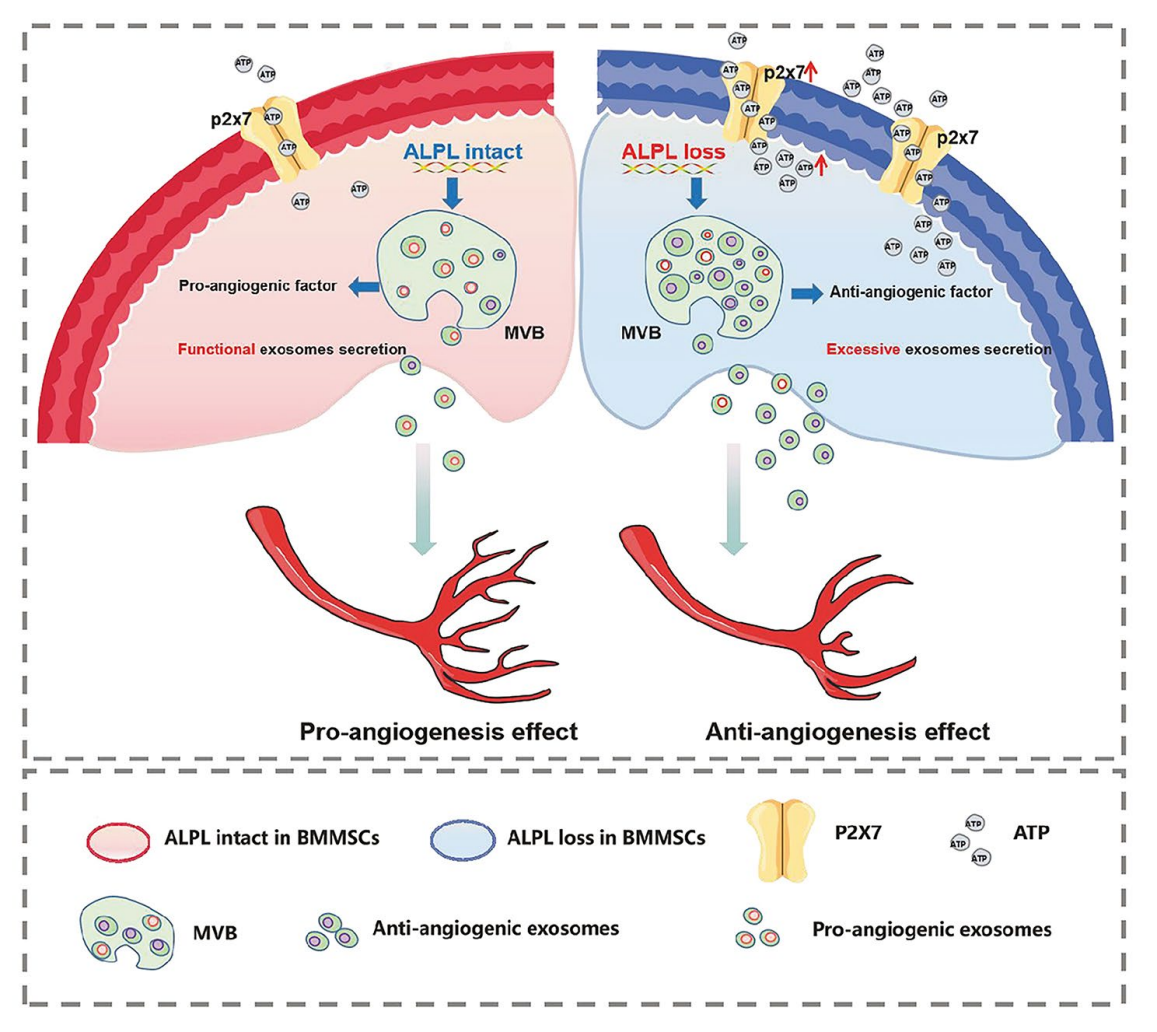
Schematic diagram depicts the detailed pro-angiogenic mechanisms of hBMMSCs exosomes mediated by ALPL-ATP axis. ALPL intact in hBMMSCs: hBMMSCs releases exosomes enriched with pro-angiogenic factors and propagates into nearby ECs. P2X7 as a major ion receptor for ATP, which mediates exosomes secretion. ALPL loss in hBMMSCs: ALPL deficiency enhances ATP release coupled with elevated expression of P2X7, resulting in excessive anti-angiogenic exosomes secretion, which in turn inhibits the angiogenic capacity of ECs.

In summary, we found that exosomes derived from hBMMSCs exhibited pro-angiogenic ability; however, ALPL deficiency in hBMMSCs resulted in increased exosomes secretion but inhibited ECs angiogenesis. This decreased pro-angiogenic capacity was caused by abnormal activation of the ALPL–ATP axis and increased P2X7 receptor expression, resulting in the secretion of more anti-angiogenic exosomes. Inhibiting the expression of P2X7 could rescue impaired angiogenesis through reducing excessive release of anti-angiogenic exosomes (Fig. 8).

## Discussion

In 1988, ALPL was discovered to encode TNSALP, and loss-of-function mutation were identified in individuals with HPP, a disease characterized by compromised bone structure, density, and strength. Our previous study suggested that ALPL facilitated bone regeneration though ATP-mediated alterations in the differentiation lineage of MSCs [4]. Angiogenesis, the development of new blood vessels from pre-existing vessels, is intimately connected with osteogenesis during the process of skeletal development and remodeling [31]. Therefore, in the current study, we propose for the first time that ALPL orchestrates hBMMSCs exosomes secretion through ATP-mediated regulation of P2X7, thereby contributing to bone angiogenesis. Specifically, ALPL deficiency results in an increase in the secretion of exosomes containing excessive anti-angiogenic cargo, which is internalized by ECs to decrease angiogenesis (summarized in Fig. 8). These findings extend the current emerging understanding of blood vessel network reconstitution during bone regeneration and could provide an alternative approach to optimize the therapeutic effects of angiogenesis by using MSCs exosomes mediated by ALPL-ATP axis.

Bone is a highly vascularized organ, and rapid and early vascularization is a prerequisite for both intramembranous and endochondral ossification during bone development [32]. MSCs are recognized as a pharmacological and therapeutic approach to facilitate angiogenesis, which can increase the growth of ECs and enhance the formation of new blood vessels [33]. MSCs can be used in the reparative process through paracrine effects that are considered to represent the predominant mechanism in recovery from tissue damage [34]. VEGF is regarded as one of the most critical factors in vascular development and regeneration [27]. Indeed, VEGF secreted from catalpol-treated BMMSCs has been shown to induce a higher level of ECs angiogenesis in the calvaria defect area of OVX rats [35]. Hence, elucidating the interaction between BMMSCs and ECs within the bone marrow microenvironment is significant for further investigation of their contribution to vascular remodeling. In bone, ALPL is localized on the entire cell surface of BMMSCs and has long been used as an osteoblast marker. hBMMSCs isolated from HPP patients exhibit extremely low ALP activity and display decreased osteogenic differentiation. Given that bone formation and pathological fracture healing is associated with modulating osteogenesis and angiogenesis, we aimed to determine whether there was a relationship between ALP activity and vascularization in bone, and to elucidate the potential mechanism. We illustrated that ALPL contributed to the promotion of ECs proliferation, migration, and tube formation via boosting BMMSCs–ECs communication, which may promote vascularization and support tissue regeneration. However, the detailed mechanisms involved in ALPL-mediated BMMSCs–ECs communication via paracrine secretion need further elucidation.

Exosomes are major mediators of cell-to-cell communication and important paracrine factors that can regulate the tissue microenvironment, encouraging tissue repair and reconstruction, and performing a broad range of biological functions. In previous studies, BMMSCs exosomes were reported to induce angiogenesis and osteogenesis in a rat nonunion model [34]. Given the role of BMMSCs in the regulation of biological function in a variety of cells via exosomes [36], it is reasonable to speculate that BMMSCs exosomes might be involved in the regulation of ALPL-mediated cell–cell communication in angiogenesis. The results of our present study suggested that ALPL regulates BMMSCs–ECs communication through exosomes secretion pathway, which indirectly orchestrates the biological behavior of ECs.

Different physiological and pathological conditions can influence the ability of exosomes to induce new vessel formation by altering their expression of both pro- and anti-angiogenic cargo [37]. In our study, we have shown that exosomes-mediated angiogenesis was related to the degree of ALPL expression, we investigated the underlying mechanisms. We found that compared with those derived from normal hBMMSCs, exosomes derived from HPP patients’ hBMMSCs markedly reduced the proliferation, migration, and tube formation of ECs, and exosomes derived from HPP patients’ hBMMSCs with recovery of ALPL expression could rescue the angiogenic capacity of ECs compared with those from HPP hBMMSCs. Importantly, exosomes secreted under conditions of ALPL deficiency in hBMMSCs contained high level of anti-angiogenic modulatory proteins such as endostatin and angiostatin. On the contrary, exosomes secreted from ALPL-upregulated in hBMMSCs had more pro-angiogenic factors such as VEGF and PDGFBB. Overall, our results suggested that the ALPL-mediated pro-angiogenic potential of hBMMSCs may be attributed to the effects of pro- and anti-angiogenic exosomes secretion.

Notably, in addition to the role of exosomes in altering expression of pro- and anti-angiogenic cargo, we found that the quantity of exosomes was the primary factor in regulating angiogenesis. Various microenvironmental factors including inflammatory cytokines, mutations, and hypoxia are known to regulate the secretion and bioactivity of MSCs exosomes [23]. Recent studies have revealed that PDGF participated in new vessels formation by stimulating MSCs after injury, which not only facilitated secretion of exosomes but also altered their proteomic content [37]. However, it is still unknown whether MSCs exosomes secretion is modulated by ALPL. Our previous studies have confirmed that bone ageing was partially orchestrated by the ALPL–ATP axis, which regulated the differentiation and senescence of MSCs [4]. It has been well established that ATP, once outside the cell, acts as an autocrine signal regulating multiple cellular functions, or as a paracrine signal that aids cell-to-cell communication [38–40]. Therefore, we propose here a mechanism by which the ALPL–ATP axis may function as a regulator of exosomes release, thereby modulating the formation of new blood vessels. Abundant evidences suggested that MSCs can release ATP spontaneously under both physiological and pathological conditions [41–43]. Moreover, extracellular ATP can, in turn, regulate MSCs differentiation, proliferation, migration, and tissue homing [44]. Here, we found that ALPL deficiency in hBMMSCs led to secretion of increased exosomes, which could be attributed to elevation of extracellular ATP concentrations. Hydrolyzation of additional extracellular ATP could rescue exosomes release in ALPL-deficient hBMMSCs. We also found that ALPL deficiency in hBMMSCs resulted in increased exosomes secretion but inhibited ECs angiogenesis, suggesting that ALPL not only regulates the release of exosomes but also alters their proteomic content. Indeed, in contrast to normal hBMMSCs, downregulation of ALPL in hBMMSCs resulted in the secretion of large numbers of anti-angiogenic exosomes and aggravated the inhibition of angiogenesis and neovascularization. According to the literature, the effects of exosomes on angiogenesis seem to be dose dependent [45]. More specifically, low concentrations of MSCs-derived exosomes (1–10 µg/mL) were found to be more effectively in promoting proliferation, migration, and tube formation than a high concentration (100 µg/mL) [46]. In the present study, we demonstrated that the low concentrations of exosomes secreted by normal hBMMSCs increased pro-angiogenic capacity, whereas high-concentration exosomes derived from ALPL-deficient hBMMSCs had inhibitory effects. Collectively, our results showed that the ALPL–ATP axis could orchestrate secretion of pro- and anti-angiogenic exosomes by hBMMSCs and had a significant impact on vascularization, supporting tissue regeneration.

ATP as a soluble factor released into the cellular matrix in response to various stimuli, which modulates signals in an autocrine or paracrine manner through specifically binding to cell surface P2X and P2Y receptors [47]. Multiple P2X and P2Y receptors have been reported in MSCs preparations from different species and tissues, albeit with some noticeable variations in receptor type [48]. In particular, the purinergic receptor P2X7, a ligand-gate ion channel, produces a diverse range of membrane trafficking responses in leucocytes and epithelial cells [49]. Evidence from previous studies suggested that P2X7 not only modulates the PI3K/AKT pathway but also acts on downstream effectors such as GSK3β, HIF-1α, and VEGF to regulate angiogenesis [50]. This evidence prompted us to further examine the functional role of P2X7 in regulating BMMSCs exosomes secretion and vascularization. We demonstrated that ALPL deficiency abnormally elevated P2X7 expression through ATP, leading to excessive release of anti-angiogenic hBMMSCs exosomes and inhibited ECs function. Furthermore, it is well-known that there is a relationship of P2X7 receptor overexpression with tumor growth, migration, and invasion, which can reduce survival and accelerate progression [50]. On the contrary, blockade of P2X7 using short hairpin RNA (shRNA) or antagonists could represent a highly effective strategy to inhibit tumor growth. Indeed, the results of our experiments indicated that the exosomes secretion by hBMMSCs with downregulation of ALPL could be rescued by P2X7 siRNA. Moreover, the angiogenic effects of ALPL-deficient hBMMSCs-derived exosomes on ECs could be ameliorated by inhibiting P2X7 expression, indicating a role of P2X7 in angiogenesis and its potential use in regeneration of bone and other tissue. At present, various P2X7 antagonists are undergoing clinical trials for the treatment of inflammatory diseases and appear to be safely tolerated by humans [51]. Nevertheless, there have been few reports of clinical trials involving blockade of the P2X7 receptor to reconstruct vascular networks in patients with pathological bone defects. Therefore, additional animal experiments are required to further examine whether P2X7 contributes to ALPL–ATP-mediated angiogenesis and bone regeneration by controlling hBMMSCs exosomes secretion. Further, a P2X7-blocking antibody to promote new blood vessel formation during bone regeneration could be tested in clinical trials.

In summary, we have demonstrated a previously unrecognized role of ALPL in vascularization involving in regulating hBMMSCs exosomes secretion mediated by ATP, and shown that P2X7 receptor blockade could represent an effective therapy for promoting angiogenesis in ALPL-deficient bone defects. The discovered mechanisms could inspired us to design much more effective treatments for HPP, for example, bioengineering particles [52, 53] that could be used to capture and clear the abnormally excess of anti-angiogenic exosomes in HPP patients, thereby rescuing impaired angiogenic capacity. Furthermore, P2X7 antagonists could represent an effective therapy to promote new vesslel formation and accelerate new bone formation by inhibiting excess exosomes secretion of hBMMSCs in HPP patients. However, the extensive application and clinical popularization of P2X7 antagonists will need further validation in animal experiments, complicated disease models and large sample sizes. Finally, studies are needed to explore whether and how ALPL–ATP-mediated pro- and anti-angiogenic cargo assembles in hBMMSCs exosomes through mechanisms involving the P2X7 receptor.

## Conclusion

In this study, we demonstrated that ALPL might be as a distinct regulator to control exosomes secretion and subsequently regulate the pro-angiogenic potential of hBMMSCs. ALPL deficiency impaired angiogenic ability of hBMMSCs, while inhibiting the expression of P2X7 could rescue such impairment through reducing the excessive secretion of anti-angiogenic exosomes. These novel insights into the mediation of exosomes release by the ALPL–ATP axis may contribute to the development of new strategies for vascularization in bone regeneration.

### Electronic supplementary material

Below is the link to the electronic supplementary material.

Supplementary Material 1

Supplementary Material 2

Supplementary Material 3

Supplementary Material 4

Supplementary Material 5

Supplementary Material 6

## Data Availability

The data supporting the findings of this study are avilable from the corresponding author upon reasonable request.

## References

[1] Li B, He X, Dong Z (2020). Ionomycin ameliorates hypophosphatasia via rescuing alkaline phosphatase deficiency-mediated L-type ca(2+) channel internalization in mesenchymal stem cells. Bone Res.

[2] Bianchi ML, Bishop NJ, Guañabens N (2020). Hypophosphatasia in adolescents and adults: overview of diagnosis and treatment. Osteoporos Int.

[3] Kusumbe AP, Ramasamy SK, Adams RH (2014). Coupling of angiogenesis and osteogenesis by a specific vessel subtype in bone. Nature.

[4] Liu W, Zhang L, Xuan K (2018). Alpl prevents bone ageing sensitivity by specifically regulating senescence and differentiation in mesenchymal stem cells. Bone Res.

[5] Liu W, Zhang L, Xuan K (2018). Alkaline phosphatase controls lineage switching of mesenchymal stem cells by regulating the LRP6/GSK3beta complex in Hypophosphatasia. Theranostics.

[6] Dong J, Zhao J, Chen J (2021). An experimental study of the role of ALPL in regulating tube formation of endothelial cells in bone marrow mesecnchymal stem cells. Pract Stomatol.

[7] Infante A, Rodriguez CI (2018). Osteogenesis and aging: lessons from mesenchymal stem cells. Stem Cell Res Ther.

[8] Diomede F, Marconi GD, Fonticoli L, et al. Functional relationship between Osteogenesis and Angiogenesis in tissue regeneration. Int J Mol Sci. 2020;21(9). 10.3390/ijms21093242. 10.3390/ijms21093242 PMC7247346 32375269

[9] Filipowska J, Tomaszewski KA, Niedzwiedzki L, Walocha JA, Niedzwiedzki T (2017). The role of vasculature in bone development, regeneration and proper systemic functioning. Angiogenesis.

[10] Ascheim DD, Gelijns AC, Goldstein D (2014). Mesenchymal precursor cells as adjunctive therapy in recipients of contemporary left ventricular assist devices. Circulation.

[11] Kourembanas S (2015). Exosomes: vehicles of intercellular signaling, biomarkers, and vectors of cell therapy. Annu Rev Physiol.

[12] Pegtel DM, Gould SJ, Exosomes (2019). Annu Rev Biochem.

[13] Rautiainen S, Laaksonen T, Koivuniemi R. Angiogenic effects and Crosstalk of adipose-derived mesenchymal Stem/Stromal cells and their extracellular vesicles with endothelial cells. Int J Mol Sci. 2021;22(19). 10.3390/ijms221910890. 10.3390/ijms221910890 PMC8509224 34639228

[14] Hu H, Zhang H, Bu Z (2022). Small extracellular vesicles released from Bioglass/Hydrogel Scaffold promote vascularized bone regeneration by transferring miR-23a-3p. INT J NANOMED.

[15] Zhang Y, Xie Y, Hao Z (2021). Umbilical mesenchymal stem cell-derived exosome-encapsulated hydrogels accelerate bone repair by enhancing angiogenesis. ACS Appl Mater Interfaces.

[16] Wu D, Kang L, Tian J (2020). Exosomes Derived from Bone mesenchymal stem cells with the stimulation of Fe3O4 nanoparticles and static magnetic field enhance Wound Healing through upregulated miR-21-5p. INT J NANOMED.

[17] Yu B, Kim HW, Gong M (2015). Exosomes secreted from GATA-4 overexpressing mesenchymal stem cells serve as a reservoir of anti-apoptotic microRNAs for cardioprotection. Int J Cardiol.

[18] Liu W, Li L, Rong Y et al. Hypoxic mesenchymal stem cell-derived exosomes promote bone fracture healing by the transfer of miR-126. *Acta biomaterialia*. 2020 02 2020;103:196–212. 10.1016/j.actbio.2019.12.020. 10.1016/j.actbio.2019.12.020 31857259

[19] Wulff K, Gatti S, Wettstein JG, Foster RG (2010). Sleep and circadian rhythm disruption in psychiatric and neurodegenerative disease. Nat Rev Neurosci.

[20] Wang K, Li Y, Ren C, Wang Y, He W, Jiang Y (2021). Extracellular vesicles as innovative treatment strategy for amyotrophic lateral sclerosis. Front Cell Dev Biol.

[21] Robbins PD, Morelli AE (2014). Regulation of immune responses by extracellular vesicles. Nat Rev Immunol.

[22] Coppi E, Pugliese A, Urbani S (2007). ATP modulates cell proliferation and elicits two different electrophysiological responses in human mesenchymal stem cells. Stem Cells.

[23] Yang R, Yu T, Kou X (2018). Tet1 and Tet2 maintain mesenchymal stem cell homeostasis via demethylation of the P2rX7 promoter. Nat Commun.

[24] Pegoraro A, Marchi ED, Ferracin M et al. P2X7 promotes metastatic spreading and triggers release of miRNA-containing exosomes and microvesicles from melanoma cells. *Cell death disease*. 2021 11 16 2021;12(12):1088. 10.1038/s41419-021-04378-0. 10.1038/s41419-021-04378-0 PMC8599616 34789738

[25] Qin Q, Lee S, Patel N (2022). Neurovascular coupling in bone regeneration. Exp Mol Med Nov.

[26] Fan Z, Liu H, Shi S (2022). Anisotropic silk nanofiber layers as regulators of angiogenesis for optimized bone regeneration. Mater Today Bio.

[27] Hu K, Olsen BR (2016). Osteoblast-derived VEGF regulates osteoblast differentiation and bone formation during bone repair. J Clin Invest.

[28] Kuang Y, Zheng X, Zhang L et al. Adipose-derived mesenchymal stem cells reduce autophagy in stroke mice by extracellular vesicle transfer of miR-25. *Journal of extracellular vesicles*. 2020 10 2020;10(1):e12024. 10.1002/jev2.12024. 10.1002/jev2.12024 PMC7710129 33304476

[29] Liu Y, Zhang Z, Wang B (2022). Inflammation-stimulated MSC-Derived small extracellular vesicle miR-27b-3p regulates macrophages by targeting CSF-1 to promote Temporomandibular Joint Condylar Regeneration. Small.

[30] Carotti V, Rigalli JP, van Asbeck-van der Wijst J, Hoenderop JGJ (2022). Interplay between purinergic signalling and extracellular vesicles in health and disease. Biochem Pharmacol.

[31] Peng Y, Wu S, Li Y, Crane JL (2020). Type H blood vessels in bone modeling and remodeling. Theranostics.

[32] Li L, Lu H, Zhao Y (2019). Functionalized cell-free scaffolds for bone defect repair inspired by self-healing of bone fractures: a review and new perspectives. Mater Sci Eng C Mater Biol Appl.

[33] Gong M, Yu B, Wang J (2017). Mesenchymal stem cells release exosomes that transfer miRNAs to endothelial cells and promote angiogenesis. Oncotarget.

[34] Zhang L, Jiao G, Ren S (2020). Exosomes from bone marrow mesenchymal stem cells enhance fracture healing through the promotion of osteogenesis and angiogenesis in a rat model of nonunion. Stem Cell Res Ther.

[35] Chen L, Zhang RY, Xie J (2021). STAT3 activation by catalpol promotes osteogenesis-angiogenesis coupling, thus accelerating osteoporotic bone repair. Stem Cell Res Ther.

[36] Reinders ME, de Fijter JW, Roelofs H (2013). Autologous bone marrow-derived mesenchymal stromal cells for the treatment of allograft rejection after renal transplantation: results of a phase I study. Stem Cells Transl Med.

[37] Lopatina T, Bruno S, Tetta C, Kalinina N, Porta M, Camussi G (2014). Platelet-derived growth factor regulates the secretion of extracellular vesicles by adipose mesenchymal stem cells and enhances their angiogenic potential. Cell Commun Signal.

[38] Huang Z, Xie N, Illes P (2021). From purines to purinergic signalling: molecular functions and human diseases. Signal Transduct Target Ther.

[39] Zhang S, Ye D, Ma L (2019). Purinergic Signaling modulates Survival/Proliferation of Human Dental Pulp Stem cells. J DENT RES.

[40] Lin Y, Nan J, Shen J (2020). Canagliflozin impairs blood reperfusion of ischaemic lower limb partially by inhibiting the retention and paracrine function of bone marrow derived mesenchymal stem cells. EBioMedicine.

[41] Dosch M, Gerber J, Jebbawi F. Mechanisms of ATP release by inflammatory cells. Int J Mol Sci. 2018;19(4). 10.3390/ijms19041222. 10.3390/ijms19041222 PMC5979498 29669994

[42] Li X, Wang X, Zhang C (2022). Dysfunction of metabolic activity of bone marrow mesenchymal stem cells in aged mice. Cell Prolif.

[43] Sun Y, Xu H, Tan B (2022). Andrographolide protects bone marrow mesenchymal stem cells against glucose and serum deprivation under hypoxia via the NRF2 signaling pathway. STEM CELL RES THER.

[44] Carluccio M, Zuccarini M, Ziberi S (2019). Involvement of P2 × 7 receptors in the osteogenic differentiation of mesenchymal Stromal/Stem cells derived from human subcutaneous adipose tissue. Stem Cell Rev Rep Aug.

[45] Todorova D, Simoncini S, Lacroix R, Sabatier F, Dignat-George F (2017). Extracellular vesicles in Angiogenesis. Circ Res.

[46] Anderson JD, Johansson HJ, Graham CS (2016). Comprehensive proteomic analysis of mesenchymal stem cell exosomes reveals modulation of Angiogenesis via Nuclear Factor-KappaB signaling. Stem Cells.

[47] Mousawi F, Peng H, Li J (2020). Chemical activation of the Piezo1 channel drives mesenchymal stem cell migration via inducing ATP release and activation of P2 receptor purinergic signaling. Stem Cells.

[48] Jiang LH, Hao Y, Mousawi F, Peng H, Yang X (2017). Expression of P2 purinergic receptors in mesenchymal stem cells and their roles in Extracellular Nucleotide Regulation of Cell functions. J Cell Physiol.

[49] Qu Y, Dubyak GR (2009). P2 × 7 receptors regulate multiple types of membrane trafficking responses and non-classical secretion pathways. Purinergic Signal.

[50] Amoroso F, Capece M, Rotondo A (2015). The P2 × 7 receptor is a key modulator of the PI3K/GSK3beta/VEGF signaling network: evidence in experimental neuroblastoma. Oncogene.

[51] Bartlett R, Stokes L, Sluyter R (2014). The P2 × 7 receptor channel: recent developments and the use of P2 × 7 antagonists in models of disease. Pharmacol Rev.

[52] Yang X, Yuan FzhouP et al. T cell-depleting nanoparticles ameliorate bone loss by reducing activated T cells and regulating the Treg/Th17 balance. *Bioactive materials*. 2021 Oct 2021;6(10):3150–3163. 10.1016/j.bioactmat.2021.02.034. 10.1016/j.bioactmat.2021.02.034 PMC7970013 33778195

[53] Bao Li D, Geng T, Ran (2022). Engineered neutrophil apoptotic bodies ameliorate myocardial infarction by promoting macrophage efferocytosis and inflammation resolution. Bioactive Mater 2022 Mar.

