# Supplementary figures and images for: ALPL regulates pro-angiogenic capacity of mesenchymal stem cells through ATP-P2X7 axis controlled exosomes secretion
Source: J Nanobiotechnology. 2024 Apr 12;22:172. doi: 10.1186/s12951-024-02396-6 (PMC11015668; doi:10.1186/s12951-024-02396-6)

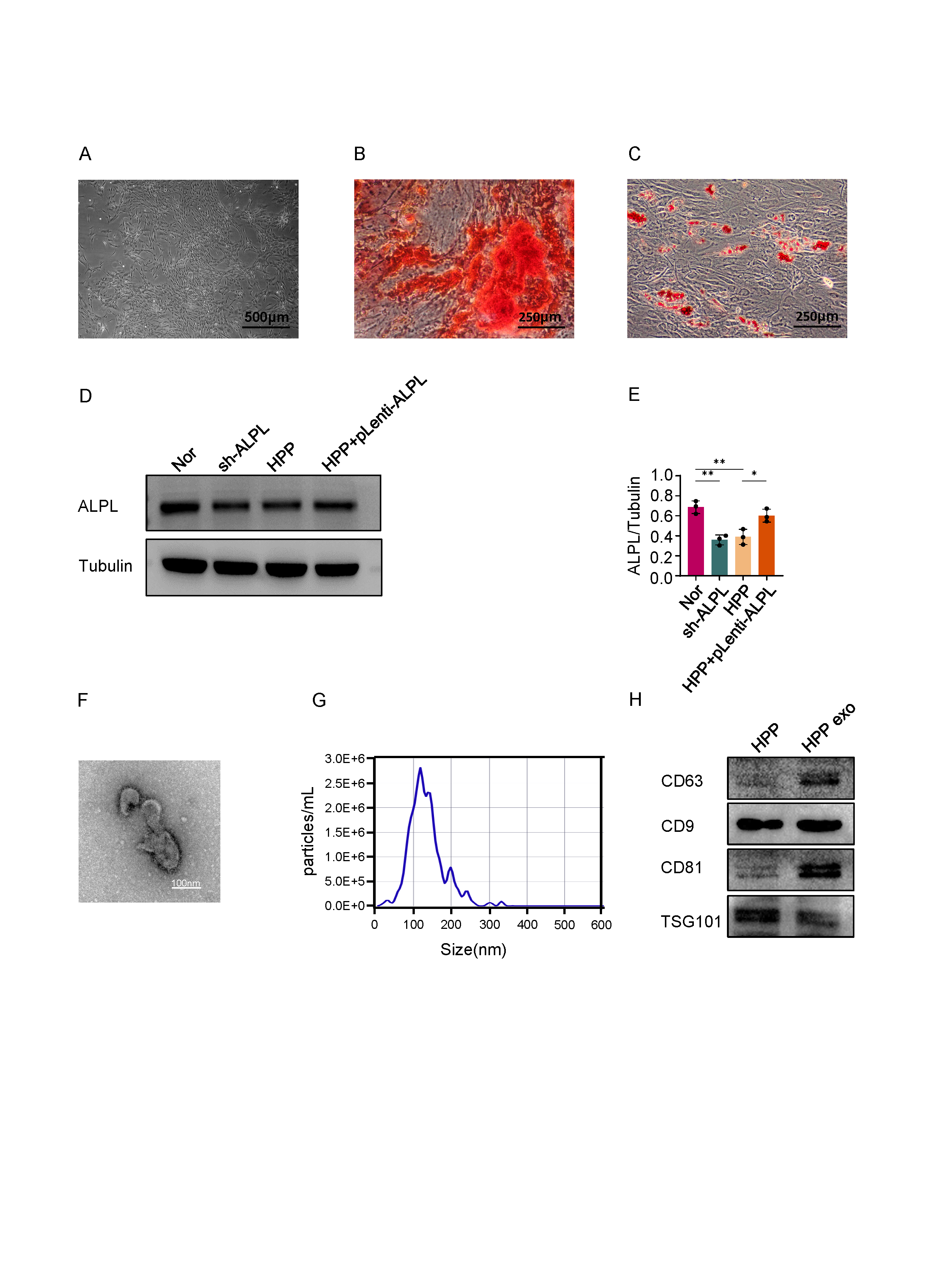

Supplement: Supplementary file 1 — Supplementary Material 1 [file 12951_2024_2396_MOESM1_ESM.png]

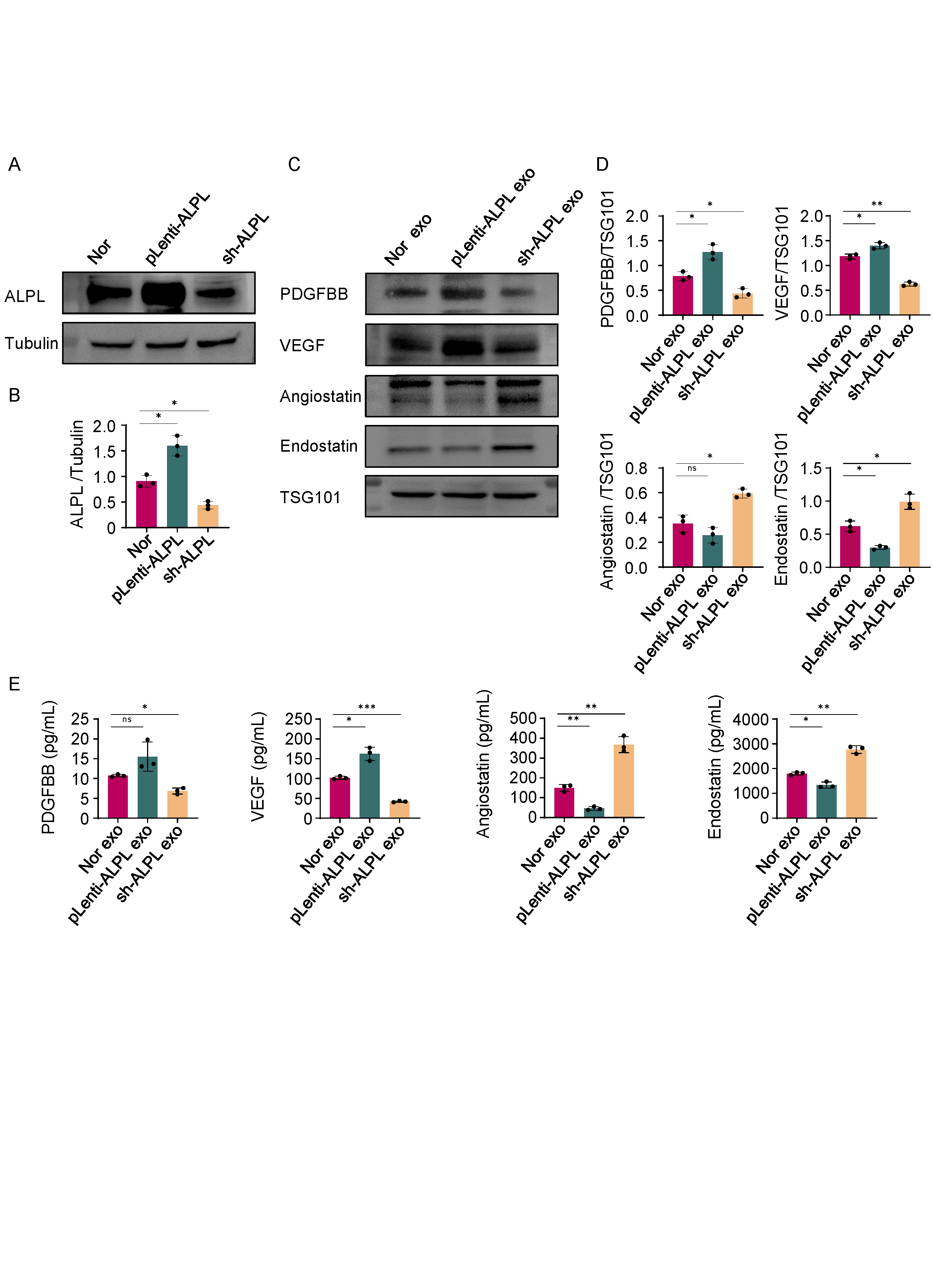

Supplement: Supplementary file 2 — Supplementary Material 2 [file 12951_2024_2396_MOESM2_ESM.png]

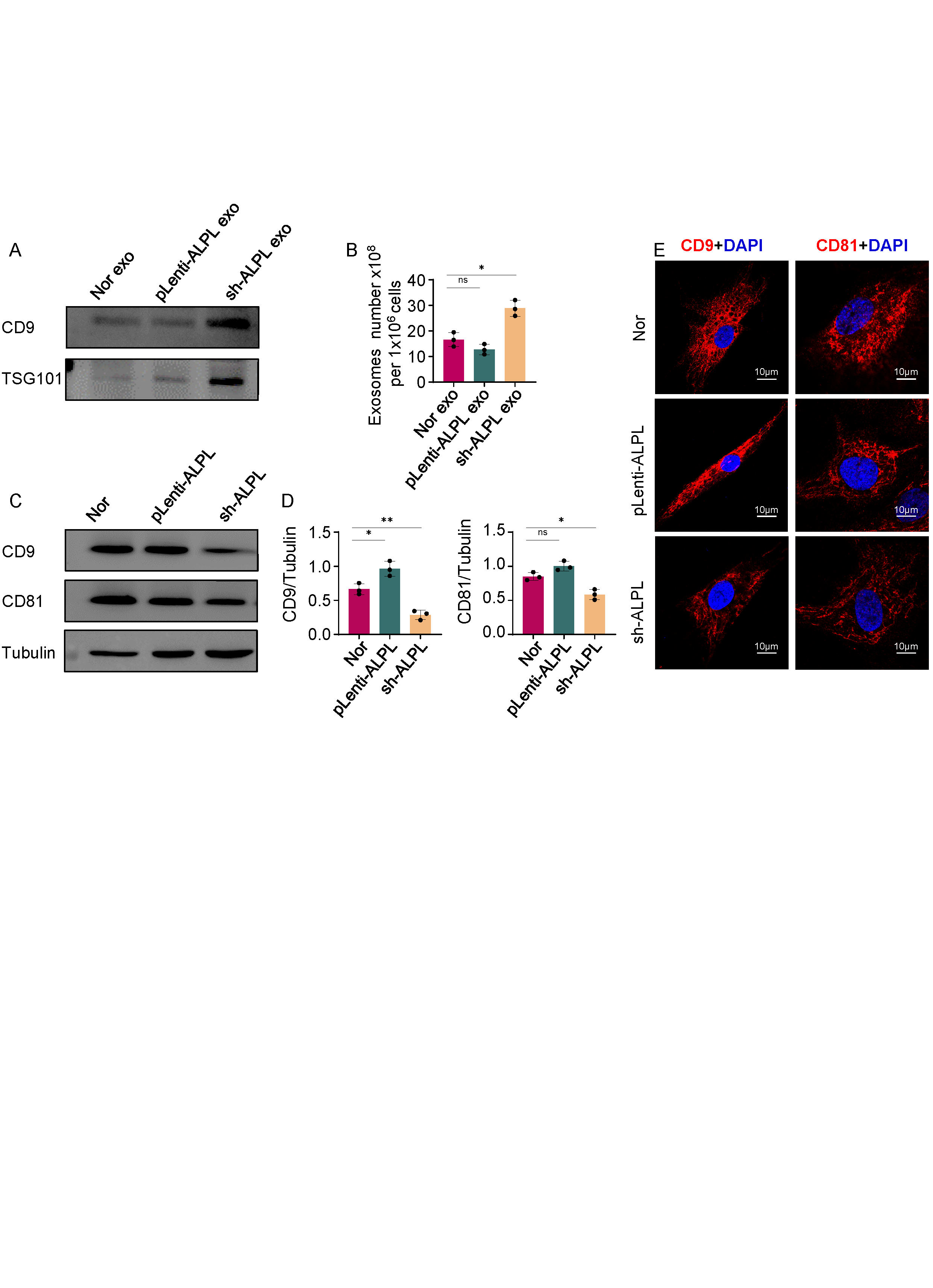

Supplement: Supplementary file 3 — Supplementary Material 3 [file 12951_2024_2396_MOESM3_ESM.png]

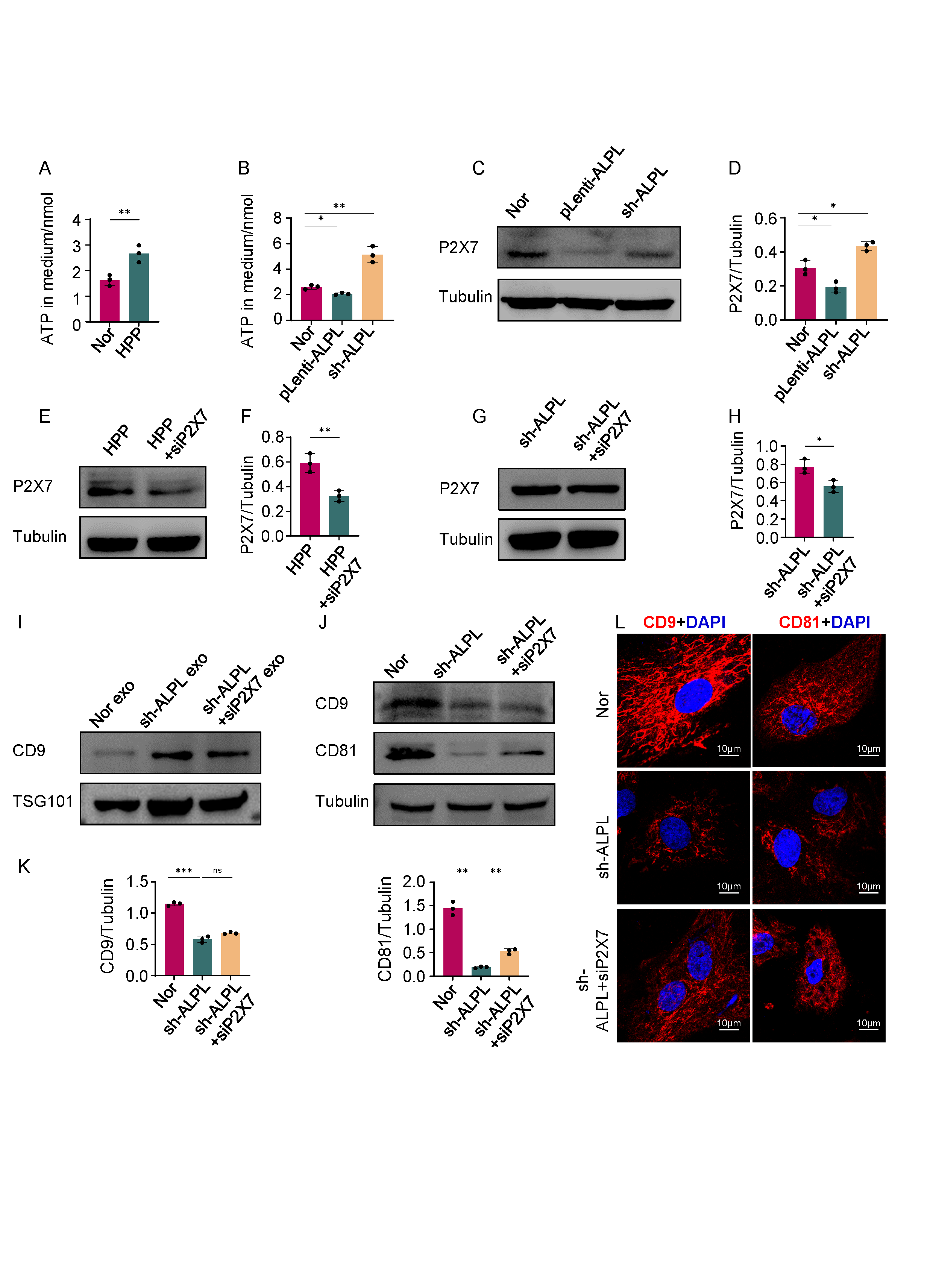

Supplement: Supplementary file 4 — Supplementary Material 4 [file 12951_2024_2396_MOESM4_ESM.png]

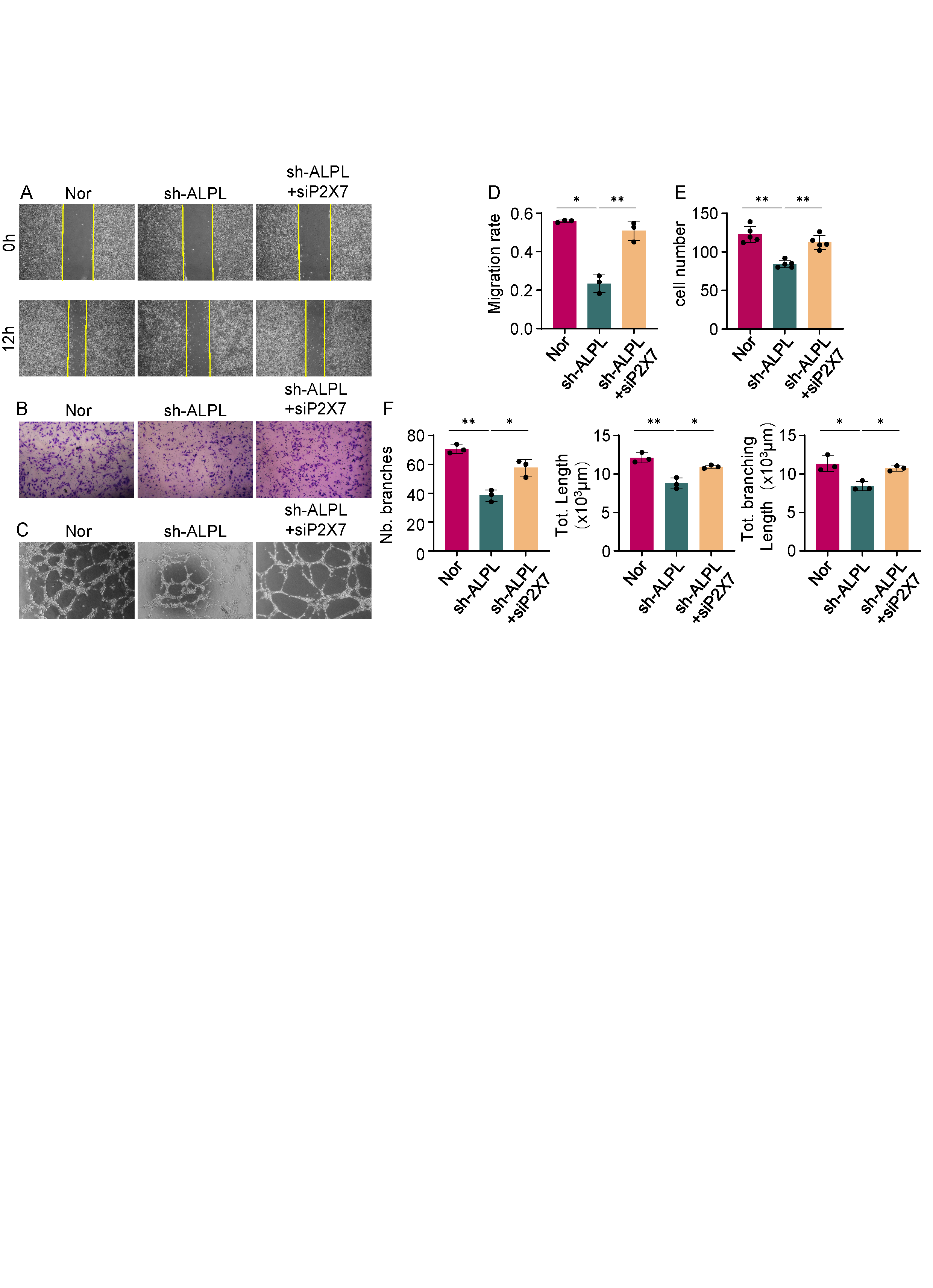

Supplement: Supplementary file 5 — Supplementary Material 5 [file 12951_2024_2396_MOESM5_ESM.png]
