# Supplementary material for: ALPL regulates pro-angiogenic capacity of mesenchymal stem cells through ATP-P2X7 axis controlled exosomes secretion
Source: J Nanobiotechnology. 2024 Apr 12;22:172. doi: 10.1186/s12951-024-02396-6 (PMC11015668; doi:10.1186/s12951-024-02396-6)
Supplement: Supplementary file 6 — Supplementary Material 6 [file 12951_2024_2396_MOESM6_ESM.docx]

Supplementary Table 1 Primers used to construct the *ALPL* lentiviral vector.

| **Name** | **Sequence** |
| --- | --- |
| ALPL shRNA | 5'-CCGGTTTGGCCAACAGGGTAGATTTCTCGAGAAATCTACCCTGTTGGCCAAATTTTT-3'  5'-AATTCAAAAATTTGGCCAACAGGGTAGATTTCTCGAGAAATCTACCCTGTTGGCCAAA-3' |
| pLenti  -ALPL | 5'-CTGGATCCGACCATGATTTCACCATTCTT-3'  5'-ATCTCGAGTGCCCTCAGAACAGGACGCT-3' |
